# A meta-epidemiological study on the reported treatment effect of pregabalin in neuropathic pain trials over time

**DOI:** 10.1371/journal.pone.0280593

**Published:** 2023-01-20

**Authors:** Emma T. L. Cheng, Mohammad Cheik-Hussein, Noelle Lin, Adriane M. Lewin, James H. McAuley, Ian A. Harris

**Affiliations:** 1 Whitlam Orthopaedic Research Centre, Ingham Institute for Applied Medical Research, Liverpool, New South Wales, Australia; 2 South West Clinical Campuses, School of Clinical Medicine, Faculty of Medicine and Health, University of New South Wales, Sydney, Australia; 3 Department of Orthopaedic Surgery, Liverpool Hospital, Liverpool, New South Wales, Australia; 4 School of Health Sciences, Faculty of Medicine and Health, University of New South Wales, Randwick, New South Wales, Australia; 5 NeuRA–Neuroscience Research Australia, Randwick, New South Wales, Australia; Keio University School of Medicine, JAPAN

## Abstract

**Background:**

Pregabalin is a drug used to treat neuropathic pain, and its use has increased substantially since 2007. Early trials found a strong treatment effect on pain for post-herpetic neuralgia and diabetic neuropathy. However more recent studies have failed to replicate these results.

**Methods:**

This meta-epidemiological study aimed to assess change in the reported effectiveness of pregabalin in neuropathic pain trials over time, and if a change is present, determine any associated factors.

**Data sources:**

We performed electronic searches for published trials in Medline, Embase and Cochrane Central Register of Controlled Trials databases; and unpublished trials on ClinicalTrials.gov, the EU Clinical Trials Register, and the Australia New Zealand Clinical Trials Registry with no restrictions.

**Study selection:**

We included randomized, placebo-controlled trials of pregabalin for treatment of neuropathic pain in adults.

**Data extraction and synthesis:**

Two authors independently extracted study data: sample size and mean baseline, end-point and change in pain scores with measures of variance, trial end year, publication year, clinical indication, funding source, country of study, treatment duration, treatment dose, mean age and percentage male.

**Primary outcome measure:**

We defined treatment effect as the mean difference in pain scores between pregabalin and placebo groups at trial end-point and assessed for change over time using a random-effects meta-regression, adjusted for sample size, indication, treatment duration (weeks) and treatment dose.

**Results:**

We included 38 randomized published trials (9038 participants) and found that between 2003 and 2020, the reported treatment effect of pregabalin decreased by 0.4 points (95% CI: 0.3 to 0.6; p<0.001) on an 11-point pain scale per 5-year interval, from 1.3 points (95% CI: 1.0 to 1.5) in trials conducted in 2001–2005, to 0.3 (95% CI: -0.1 to 0.7) in trials conducted in 2016–2020. The reported treatment effect was lower than the minimal clinically important difference (MCID) of 1.7 points across all time periods, doses and most indications and was not found to be associated with study characteristics.

**Conclusions:**

The reported treatment effect or analgesic efficacy of pregabalin from clinical trials has diminished over time. Clinical recommendations may need to be re-evaluated to account for recent evidence and to consider whether pregabalin therapy is indicated.

## Introduction

Pregabalin (Lyrica) is used to treat neuropathic pain [1–3], a debilitating condition caused by disease to the somatosensory system that is often refractory to treatment [4]. Since its introduction the use of pregabalin has grown internationally, with gross sales in 2017 estimated at US$5.1 billion [5]. In the UK pregabalin prescribing increased by 350% between 2007 and 2012 [6]. In Australia, prescriptions increased from 140,000 in 2013, when pregabalin became government subsidized, to 3.2 million in 2020; the total cost in 2020 was AU$104 million [7, 8].

Early pregabalin trials found a strong treatment effect on pain intensity in postherpetic neuralgia (PHN) and diabetic peripheral neuropathy (DPN) [9–12], however recent studies have failed to replicate these effects [13–16]. This decrease in reported treatment efficacy over time in research trials, known as the decline effect [17], may be associated with changes in study population, indications, dosage, outcome measures, treatment duration or study quality. A decline effect has been shown in psychology research, and pharmacological and vertebroplasty trials [18–21]. A 2018 study by Finnerup et al. demonstrated decreasing estimated efficacy of various drugs in neuropathic pain trials [22].

This meta-epidemiological study of randomized controlled trials (RCTs) aimed to:

- identify any change over time in the treatment effect (analgesic efficacy) of pregabalin compared to placebo for neuropathic pain, using meta-regression, and, if present;
- determine factors associated with any change in treatment effect.

## Methods

### Search strategy and reporting

This meta-epidemiological study was prospectively registered on PROSPERO (https://www.crd.york.ac.uk/prospero/; Record ID CRD42018106925) and reported using the “Guidelines for reporting meta-epidemiology methodology research”, adapted from PRISMA guidelines [23].

### Eligibility criteria

We included published and unpublished RCTs of pregabalin for treatment of neuropathic pain in adults (≥18 years). Studies reporting pain scores on an 11-point Numeric Rating Scale [NRS; range: 0 (“no pain”) to 10 (“worst possible pain”)] were eligible for inclusion. Studies using a 0–100 visual analogue pain scale were included after rescaling (dividing by 10). Neuropathic pain was defined as "Pain caused by a lesion or disease of the somatosensory system" (International Association for the Study of Pain) [24]. This includes, for example, DPN, PHN, HIV-associated neuropathy, central neuropathic pain, post-traumatic peripheral neuropathy and sciatica. We excluded trials where neuropathic pain was not established (i.e. experimental studies in healthy volunteers), prophylactic or peri-operative pregabalin use, and other indications including epilepsy and fibromyalgia.

Studies were eligible if data from placebo and pregabalin groups could be extracted separately. Data from unpublished trials were obtained directly from trials registry websites. Enriched-enrolment trials, which use a single-blind run-in period prior to randomization to identify and exclude both placebo responders (pain reduction ≥ 50%) and pregabalin non-responders (pain reduction ≤ 30%), were included in sensitivity analyses only as they may be biased towards higher estimates of treatment effect [25]. We excluded studies allowing additional concomitant pain treatment unless a stable dose had been reached at least one week before study commencement.

### Information sources and search strategy

We performed electronic searches for published trials in Medline, Embase and Cochrane Central Register of Controlled Trials databases; and unpublished trials on ClinicalTrials.gov, the EU Clinical Trials Register (clinicaltrialsregister.eu), and the Australia New Zealand Clinical Trials Registry (anzctr.org.au) with no time or language restrictions. We hand-searched reference lists of identified articles. The search was completed in October 2022. The full search strategy is included in S1 Table.

### Study selection

For published trials, two authors (EC, MCH) independently conducted title-and-abstract screening, followed by full-text review to determine study eligibility. For unpublished studies, two authors (MCH, AML) assessed study details available on trial registries against eligibility criteria. Disagreements were resolved through discussion.

### Data extraction process and data items

Two authors (EC, MCH) independently extracted study data including sample size (number randomized), mean baseline, end-point and change in pain scores with measures of variance (standard deviation [SD], standard error), publication year (published studies only), trial end year, approval year, indication (reason for treatment), funding source, country of study, trial duration (weeks), treatment duration (weeks), treatment dose, mean age and percentage male. For crossover trials, pain scores from the first treatment period were used.

### Risk of bias

Two investigators (EC, AML) independently assessed individual studies using the Cochrane Risk of Bias tool [26], with discrepancies resolved through discussion.

### Primary outcome

The primary outcome was the change in reported treatment effect of pregabalin over time, presented in 5-year periods for ease of interpretation. The treatment effect of pregabalin was defined as mean between-group difference in end-point pain scores (placebo minus pregabalin; a positive score favors pregabalin). The accepted minimal clinically important difference (MCID) for chronic pain, i.e., the smallest difference that is meaningful for patients, is a reduction of at least 1.7 points in average pain severity on a 11-point scale [27–32].

### Publication bias

We assessed publication bias using a funnel plot and Egger’s regression [33, 34].

### Data analysis

All outcomes were analyzed using trial-level, intention-to-treat data. To assess the treatment effect of pregabalin using meta-analysis, we combined end-point pain scores across trials using an inverse-variance weighted random-effects model to give an overall mean difference in end-point pain scores between pregabalin and placebo groups. We evaluated between-study heterogeneity using I^2^ [35], Cochran’s Q statistic [36], and tau^2^. We used the DerSimonian-Laird estimator for tau^2^ with Hartung-Knapp-Sidik-Jonkman adjustment [37–40].

Where studies reported only baseline and change scores, we calculated end-point pain scores using the following assumptions: between-group SDs were the same across studies; and within-group SDs in change scores and end-point scores were the same in each study arm [41]. For studies assessing multiple pregabalin doses, we pooled end-point pain scores of the pregabalin groups to yield a single comparison group for the primary analysis [42].

To assess whether the treatment effect of pregabalin changed over time, we used random-effects meta-regressions with mean between-group difference as the dependent variable and publication year as the independent variable. We adjusted for sample size, indication, treatment duration and treatment dose. For models assessing outcome by pregabalin dose, the end-point pain scores of the control group were split to match the number of pregabalin groups to avoid unit-of-analysis error [42]. We performed meta-analyses on subgroups defined by indication, dose and risk of bias domains, and performed a network meta-analysis by dose.

We performed three sensitivity analyses including either unpublished or enriched-enrolment trials in the meta-analysis and meta-regression, and also with publication year grouped into quartiles rather than 5-year periods to distribute trials evenly within each quartile.

We used Spearman’s rank-order correlation to assess the relationship between publication year and sample size, treatment duration, and treatment dose. We used Fisher’s exact test to assess the relationship between publication year and indication, and each Cochrane risk of bias domain. We used Pearson’s correlation to assess the within-group association between year and baseline and end-point pain scores. Analyses were conducted using R version 4.0.2 [43].

### Role of funding source

There was no funding source for this study.

## Results

### Study selection and characteristics

**Fig 1:**
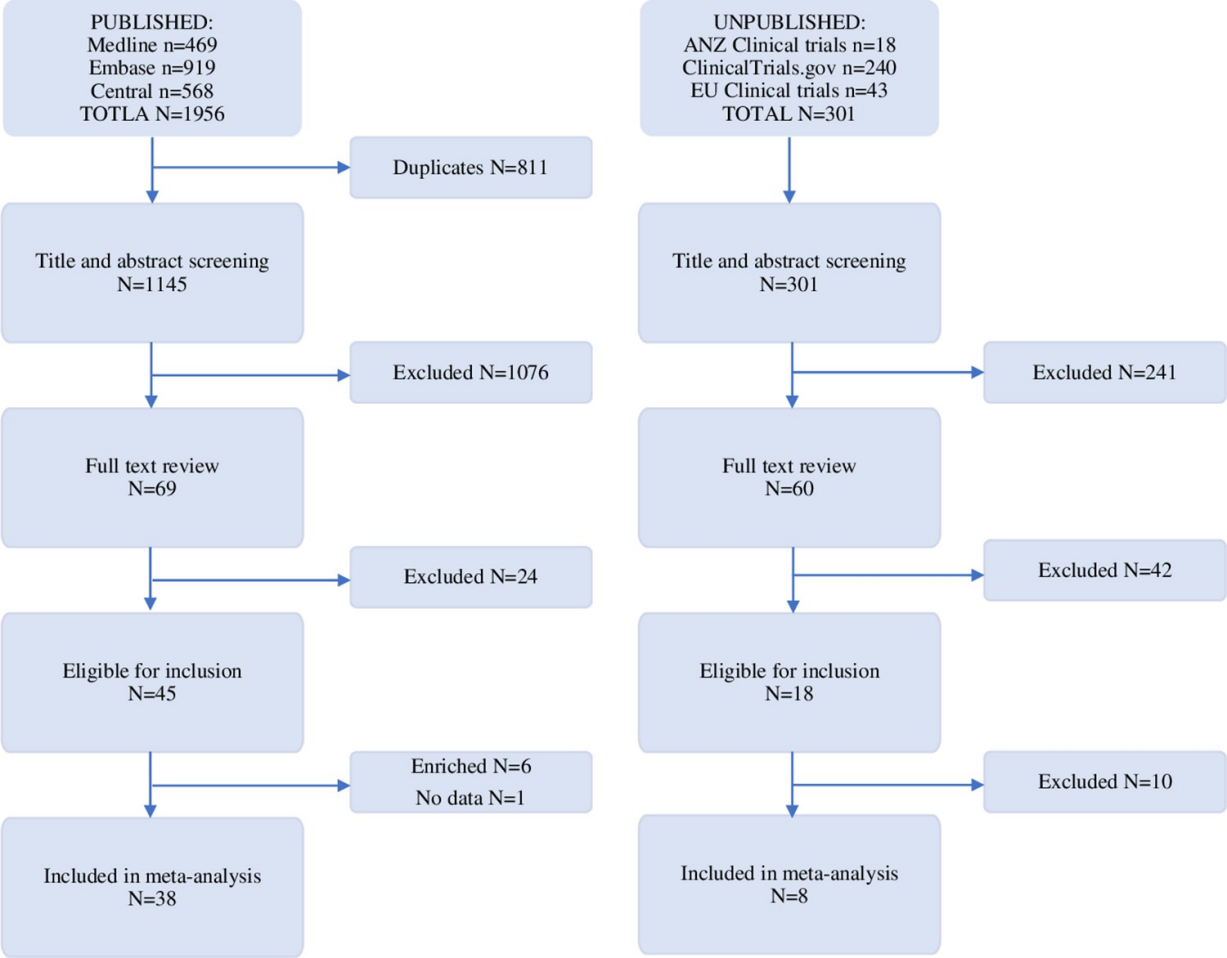
Study selection.

We identified 1,956 published and 301 unpublished RCTs. After exclusions 38 published trials (n = 9038 participants) were included in our primary analysis (Fig 1). Eight unpublished (n = 2294 participants) and 6 enriched-enrolment trials (n = 1210) were included in sensitivity analyses. Among published trials, 42.1% (n = 16) were conducted for treatment of diabetic polyneuropathy and 13.2% (n = 5) for post-herpetic neuropathy. Sample size ranged from 19 to 620 participants (mean = 237; SD = 154) and mean age of participants was 57.6 (SD = 7.3) years. Among unpublished trials, 25.0% (n = 2) were conducted for treatment of diabetic polyneuropathy, 25.0% (n = 2) for post-herpetic neuropathy, and 25.0% (n = 2) for central neuropathic pain. Sample size ranged from 29 to 620 participants (mean = 287; SD = 211) and mean age of participants was 61.5 (SD = 5.8) years. Included published studies are described in S2 Table; identifiers for unpublished studies are shown in S3 Table.

### Assessment of study quality

**Fig 2:**
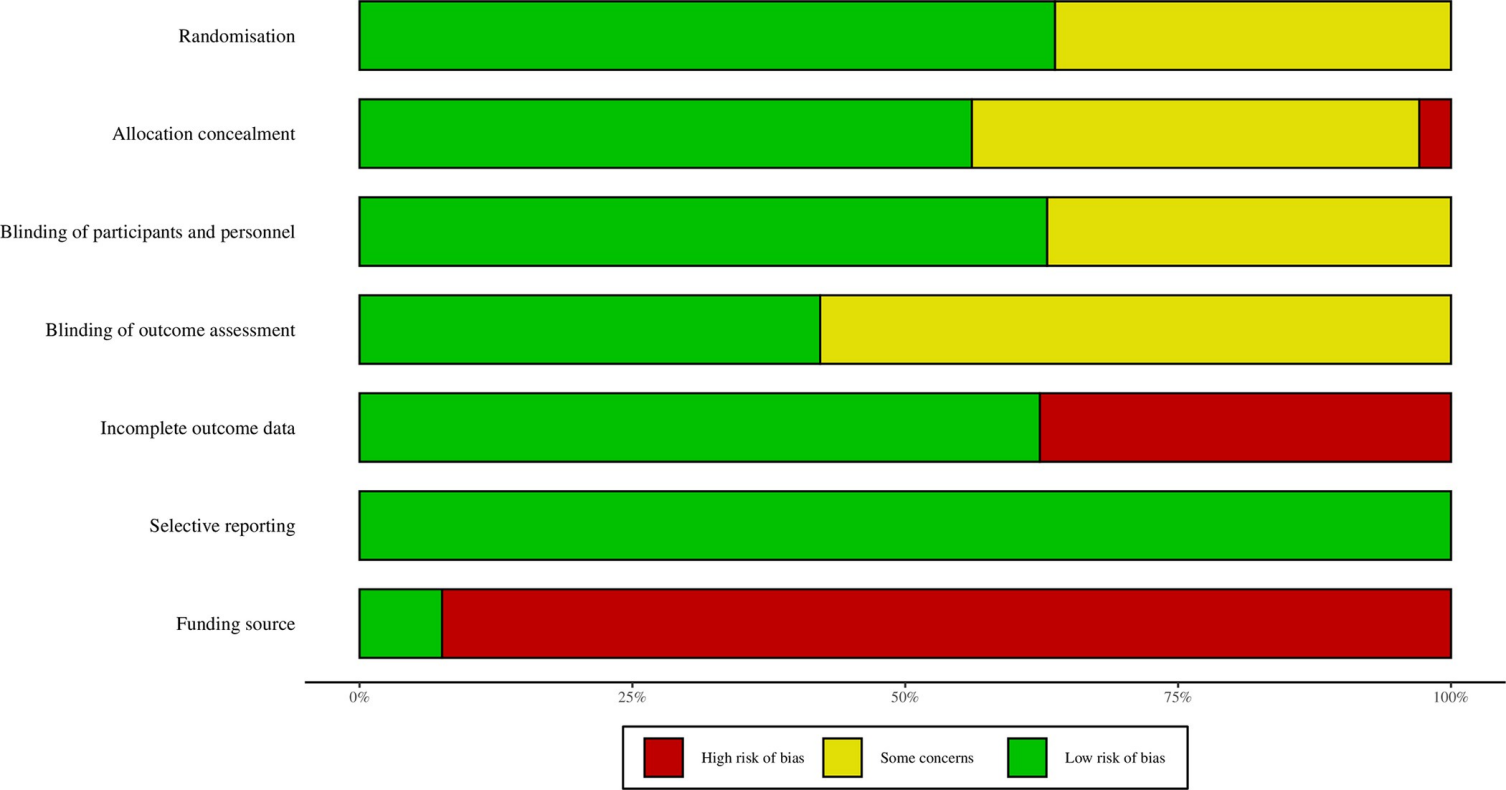
Cochrane risk of bias assessment of 37 trials published in English*. * One trial by Ogawa et al. (2010) excluded [44].

We assessed risk of bias (ROB) in the 37 trials published in English. ROB was high for the domain “funding source”, as 32/37 studies (86%) were funded by a pharmaceutical company. Attrition bias was also high: 14/37 (38%) studies reported incomplete outcome data. ROB was moderate for the remaining domains (Fig 2).

### Meta-analysis: Treatment effect of pregabalin overall and by publication year

Mean (SD) pain scores were 6.6 (0.4) and 6.5 (0.4) in the placebo and pregabalin groups at baseline, and 5.0 (0.8) and 4.4 (0.6) at end-point, respectively, with an overall between-group difference in pain reduction (or reported treatment effect) of 0.6 points (95% confidence interval [CI]: 0.4 to 0.8), favoring pregabalin.

**Fig 3:**
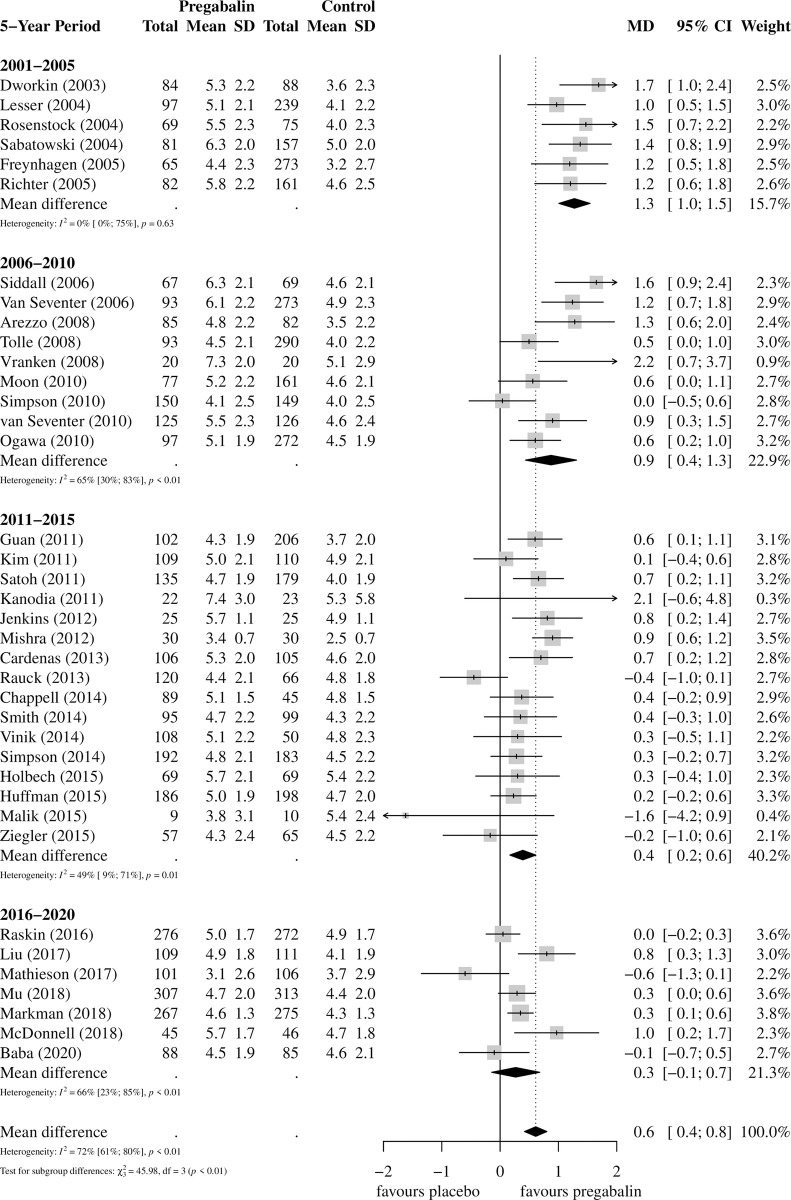
Forest plot of reported treatment effect* of pregabalin by 5-year interval and overall. * Treatment effect defined as: mean difference (MD) in end-point pain scores between pregabalin and placebo. Abbreviations: SD, standard deviation; MD, mean difference; 95% CI, 95% confidence interval.

The reported treatment effect was greater in earlier trials compared to more recent trials but was less than the MCID of 1.7 points in all time periods: 1.3 points (N = 6 trials; n = 1471 participants; 95% CI: 1.0 to 1.5) in 2001–2005 and 0.3 (N = 7 trials; n = 2401 participants; 95% CI: -0.1 to 0.7) in 2016–2020 (Fig 3). Subgroup analyses by indication and dose and network meta-analysis of doses found the treatment effect was less than the MCID for all doses and indications, except for acute herpetic neuralgia (2.1, 95% CI: -0.6 to 4.8; 1 trial; 45 participants) (S1–S3 Figs).

### Change in treatment effect over time: Meta-regression of treatment effect on publication year

**Fig 4:**
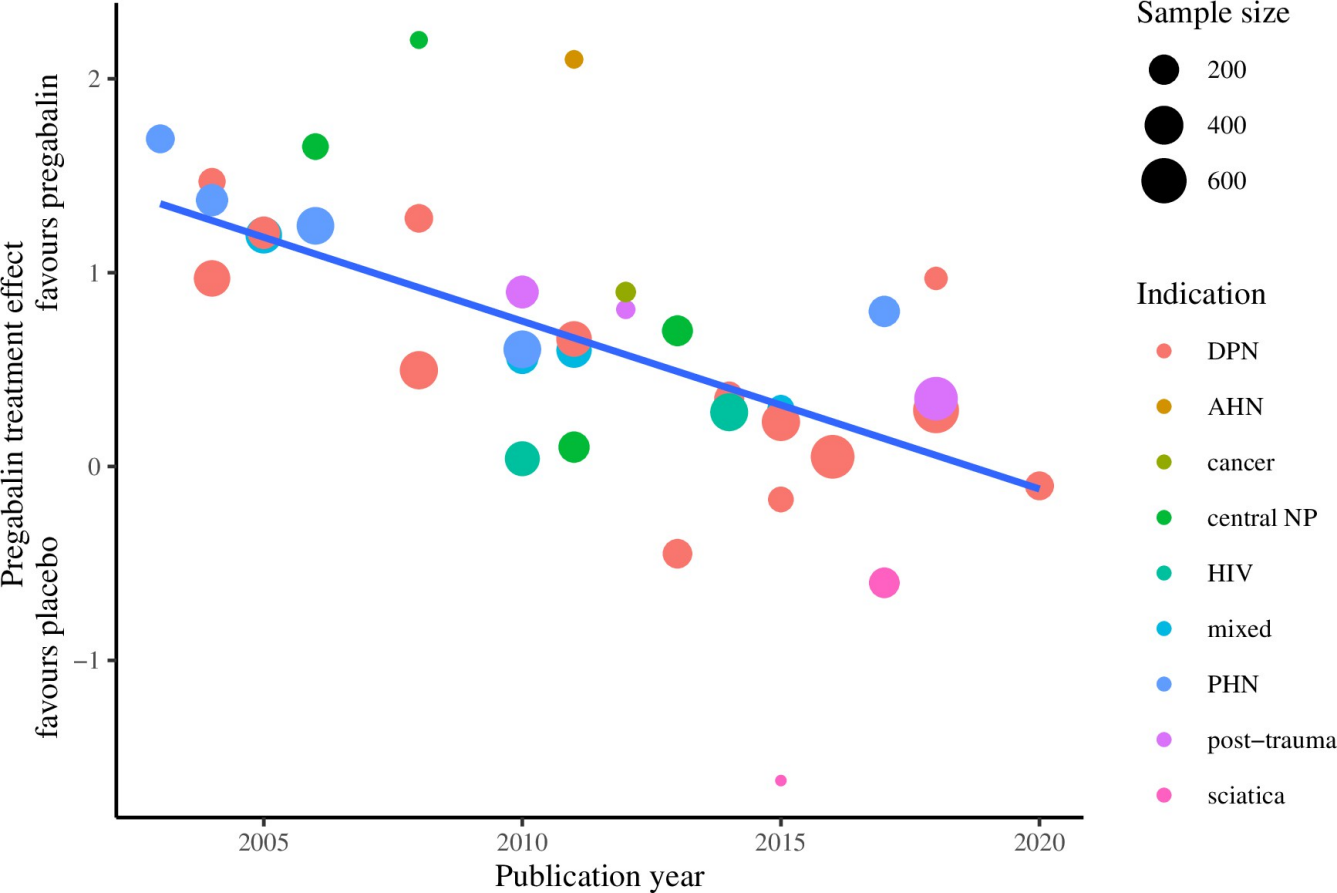
Treatment effect of pregabalin over publication year* overall. * Derived from unadjusted meta-regression models. Subgroups with two or fewer studies were not evaluated.

**Fig 5:**
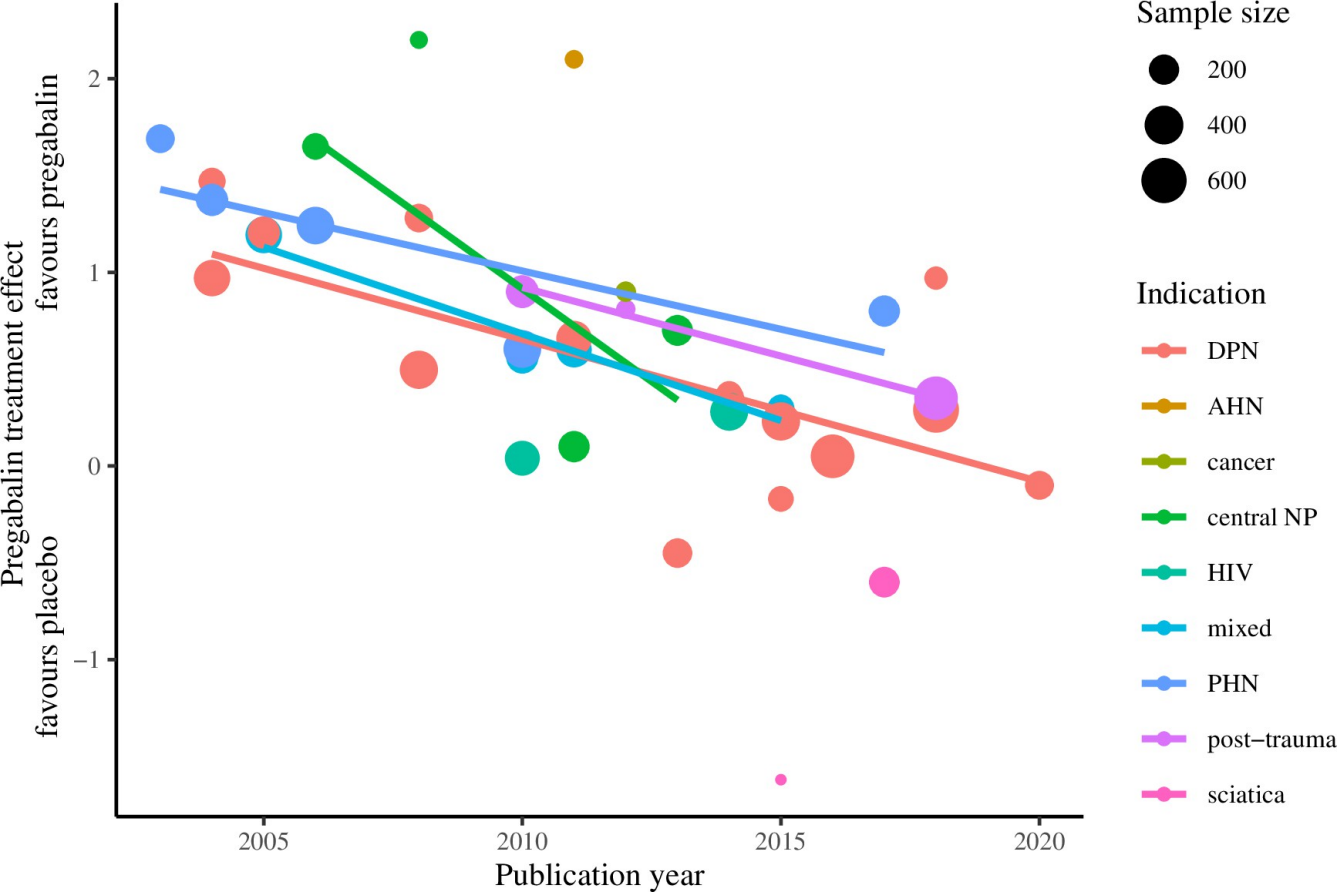
Treatment effect of pregabalin over publication year* by indication. * Derived from unadjusted meta-regression models. Subgroups with two or fewer studies were not evaluated.

**Fig 6:**
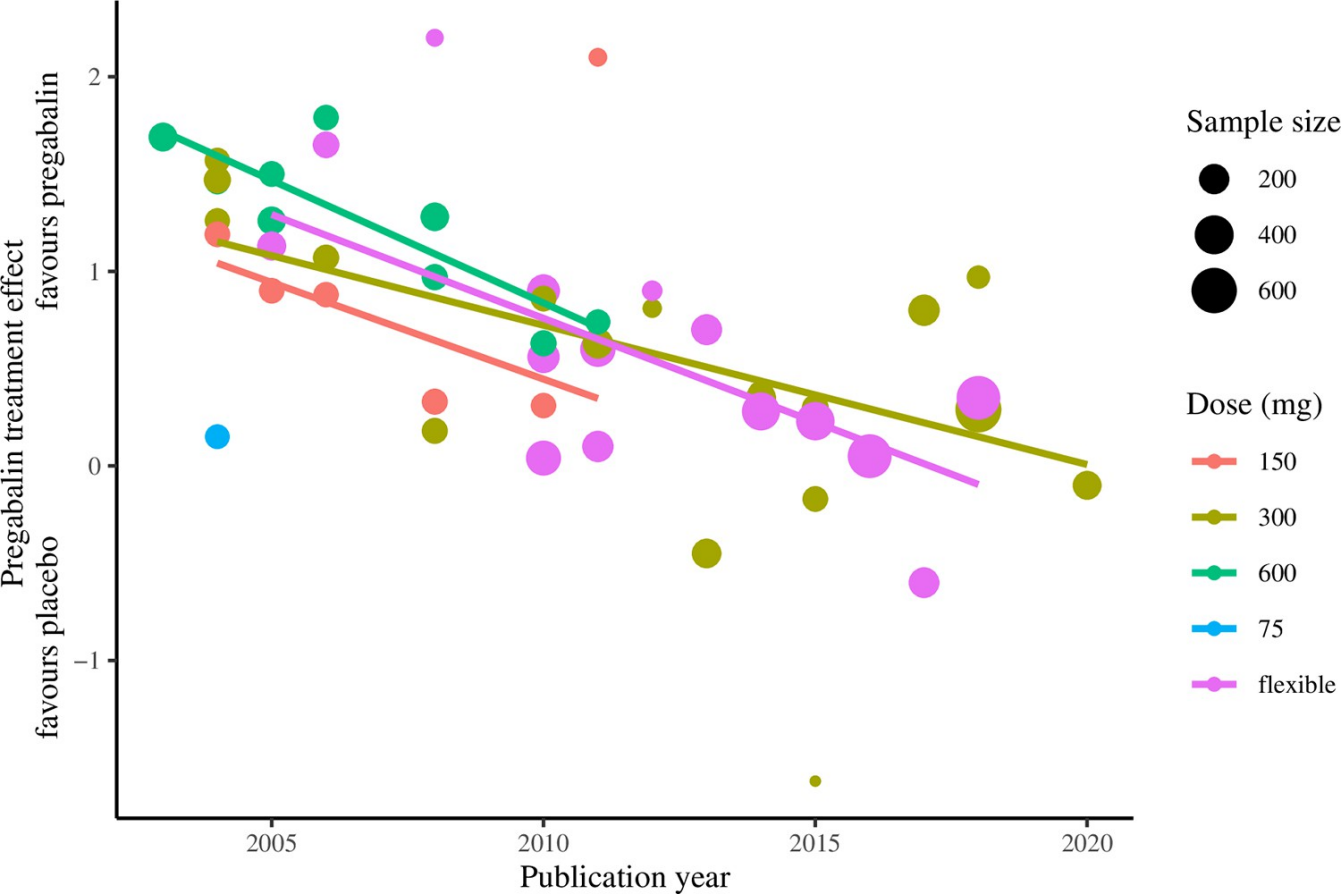
Treatment effect of pregabalin over publication year* by dose. * Derived from unadjusted meta-regression models. Subgroups with two or fewer studies were not evaluated. Abbreviations: DPN, diabetic peripheral neuropathy; AHN, acute herpetic neuralgia; central NP, central neuropathic pain; HIV, human immunodeficiency virus-associated neuropathy; PHN, post-herpetic neuralgia.

A decline in treatment effect was observed with increasing publication year. In an unadjusted meta-regression estimating effect size by publication year, treatment effect decreased by 0.1 points every year, or 0.4 points every 5 years (95% CI: 0.3 to 0.6; p<0.001) (Table 1; Fig 4). Results were similar after adjustment for sample size, indication and treatment duration, with a decrease in treatment effect of 0.3 points per five-year interval from 2001–05 to 2016–20 (95% CI: 0.2 to 0.5; p<0.001). Multivariable-adjusted models stratified by indication and dose returned similar results (Table 1; Figs 5 and 6). Subgroups with two or fewer studies (HIV, sciatica and APN) were not evaluated.

**Table 1:** Meta-regression results for the association between treatment effect size and year for unadjusted, partially- and fully-adjusted models.

Model	Pregabalin treatment effect size (points) per 5-year period (95% CI)
Unadjusted (effect of year alone)	-0.4 (-0.6 to -0.3)
Adjusted for indication only	-0.4 (-0.5 to -0.2)
Adjusted for indication, treatment duration (weeks) and study sample size	-0.3 (-0.5 to -0.2)
Adjusted for dose* only	-0.4 (-0.6 to -0.3)
Adjusted for dose, indication, treatment duration (weeks) and study sample size	-0.4 (-0.5 to -0.2)

Abbreviations: CI = confidence interval; N = sample size; yr = publication year.

### Sensitivity analyses

Sensitivity analyses including unpublished or enriched-enrolment returned results similar to the primary analysis, as did using study publication year in quartiles (S4–S8 Figs; S4 Table).

#### Influence of study characteristics on treatment effect of pregabalin over time

Publication year was poorly correlated with (Fisher’s exact test, p = 0.88), sample size (Spearmans rho: -0.03), indication treatment duration (Spearmans rho, -0.1) and treatment dose (Spearmans rho, -0.1; S5 and S6 Tables).

While attrition bias appeared to decrease over time and inadequate blinding of participants and personnel increased, no associations were found between risk of bias domains and publication year (S7 Table, S9 Fig). Additional subgroup analyses by risk of bias domain, restricted to domains with sufficient distribution of studies (K>10), found no difference in treatment effect of pregabalin between studies at low risk of bias vs unclear/high risk of bias (S10 Fig).

#### Converging pain score measurements over time

**Fig 7:**
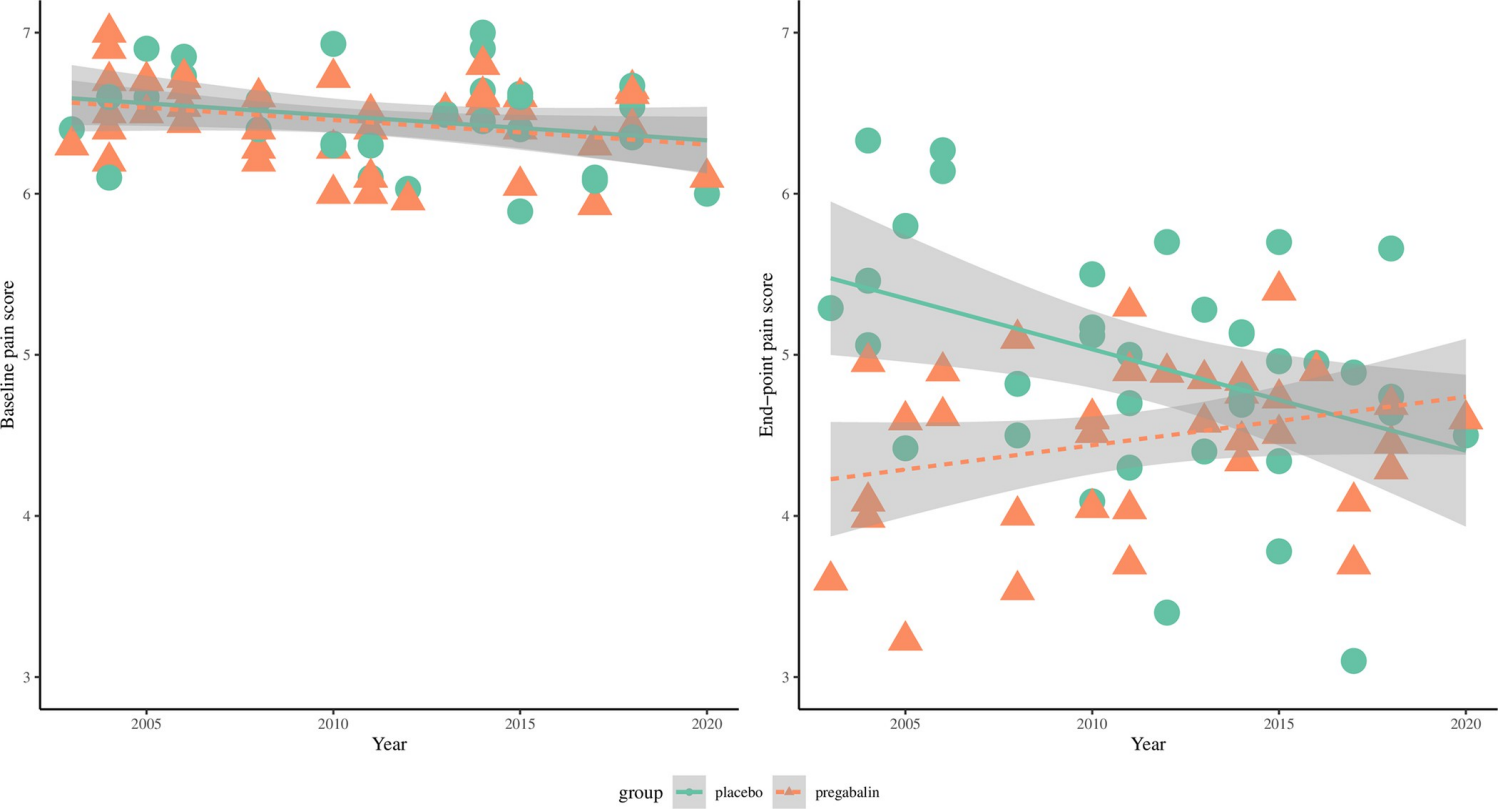
Baseline and end-point pain scores in the pregabalin and placebo groups, 2003–2020. Pearson’s correlation (r) for change in within-group pain scores over publication year at baseline: placebo, r = -0.20; pregabalin, r = -0.15; at end-point: placebo, r = -0.38; pregabalin, r = 0.22.

Baseline pain scores were similar in both groups and changed little by year. End-point pain scores decreased over time (i.e. better pain reduction) in the placebo group with no corresponding change in the pregabalin group (Fig 7).

### Publication bias

**Fig 8:**
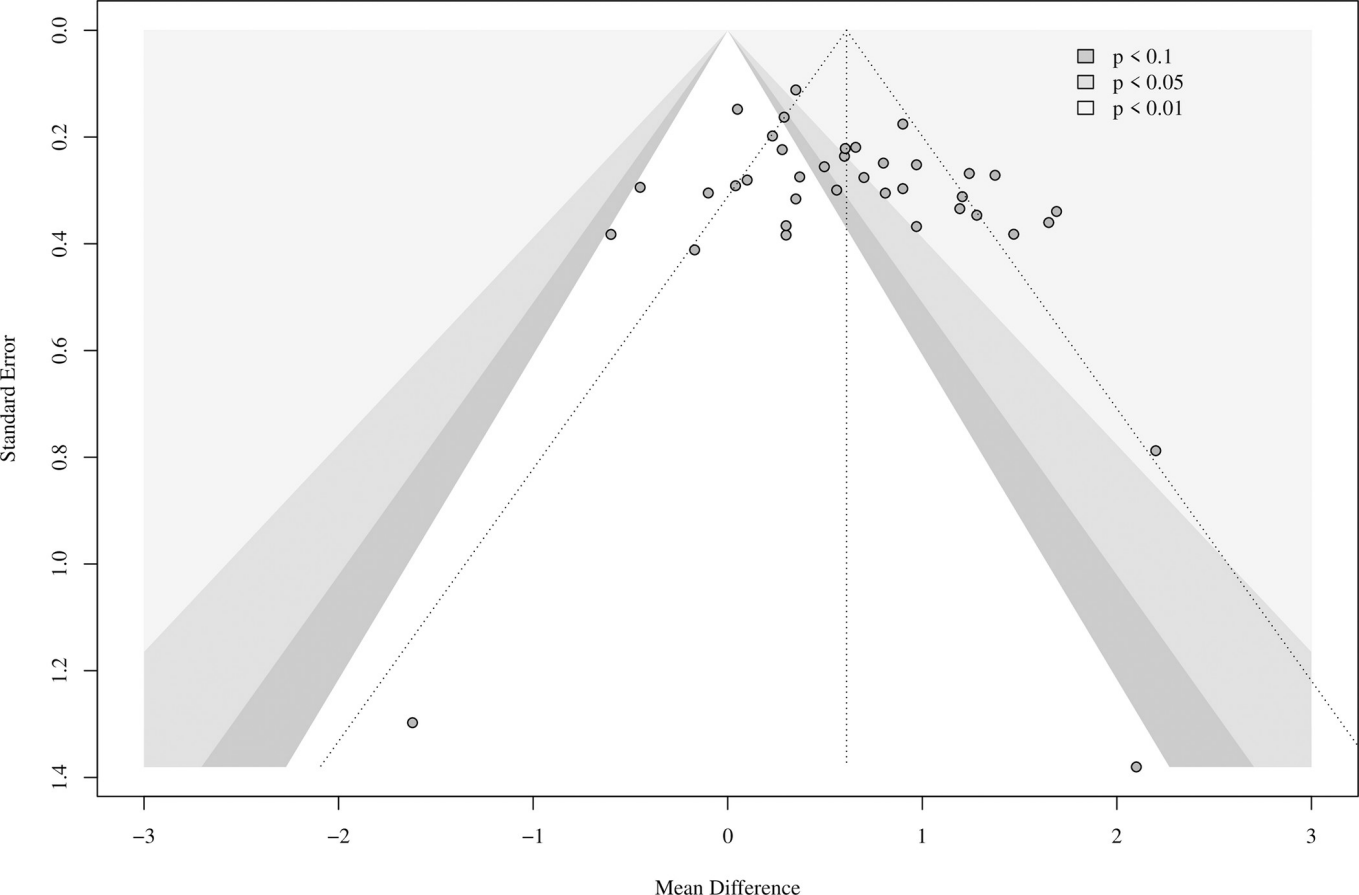
Funnel plot of published trials.

There was no evidence of publication bias (Fig 8). The studies are distributed symmetrically around the pooled effect size (dotted line); Eggers’ test for asymmetry was insignificant (p = 0.09).

## Discussion

### Summary of main findings

This meta-epidemiological study of RCTs found pregabalin was associated with a small reduction in pain relative to placebo and identified a decline in the reported treatment effect of pregabalin for neuropathic pain over time, which persisted across all doses and indications and was robust to three sensitivity analyses. In all analyses, the treatment effect was below the minimum clinically important difference. The decline in effect size over time was not associated with any study or participant characteristics, or risk of bias domains. While baseline pain scores remained similar in both groups, there was significant improvement in trial-end pain scores in the placebo group.

### Interpretation

The decline in treatment effect over time likely does not reflect a true change in the efficacy of pregabalin. Rather, it may reflect the use of pregabalin in different populations or for different types of neuropathic pain, the use of co-treatments, or improved study quality. Two prior systematic reviews reported larger sample size, longer study duration and certain participant characteristics, such as increased age, male sex and higher baseline pain intensity as well as the pain condition, specifically DPN, were associated with decreased pain in the placebo group [22, 45, 46]. This may reflect an underlying spectrum bias [19], where the difference in drug effect is related to the range of individuals included in different studies. Nevertheless, these findings were not replicated in our study: sample size, duration, proportion of male participants and baseline pain intensity remained constant over time, while mean age decreased; additionally, the decline in treatment effect was observed for all indications except acute herpetic neuralgia, HIV-associated neuropathic pain and sciatica, where there were insufficient number of studies to establish a trend. As such, decreasing end-point pain scores in the placebo group were not associated with any study or participant characteristics, baseline differences in the study population, or the clinical indication. Improved co-treatments potentially contributed to improved pain reduction in the placebo group, leading to an underestimation of the treatment effect; included trials permitted concomitant use of neuropathic pain treatment (including opioids if at a stable dose at least one week before study commencement). It is possible that as pregabalin became more commonly prescribed and known, participant expectation and belief in the benefit of pregabalin could explain the increase in placebo response in later trials. However, we were not able to assess this in the current study. The decline effect may be associated with publication bias and changes in study quality [19, 47]. While publication bias can result in delayed publication of negative results, the decline effect persisted in a sensitivity analysis including unpublished trials. Improvement in study quality over time may result in smaller effect sizes through diminished placebo response [48]. There is some evidence of decreasing attrition bias over time, which may have contributed to the decline effect observed here [21]. However, our study was underpowered to detect a difference in sub-group analyses. While publication bias and improvement in allocation concealment and blinding were unlikely to have contributed to the decline effect, we could not exclude attrition bias as a possible explanation.

### Compare & contrast with other studies

A review of pharmacological treatments for neuropathic pain reported an increase in the number needed to treat with pregabalin from 4 to 7.7 between 2003 and 2017 [22], i.e., 35% of participants in the pregabalin and 24% in the placebo group experienced a 50% reduction in pain intensity [49]. Similar to our findings, authors reported decreasing end-point pain scores over time in the placebo group; however it was associated with larger sample sizes and longer study duration, which were not supported by our findings based on pregabalin trials alone.

The decline effect has been observed in other drug therapies. Gehr (2006) analyzed RCTs between 1978 and 2001 and found a decline effect for pravastatin, timolol and latanoprost, but not for atorvastatin, possibly as the latter was relatively new at the time. In their study, baseline values were the strongest predictor of outcome, which they attributed to spectrum bias. They also found that sample size increased over time and drugs changed from the experimental to the control group. In our study, we found no changes in baseline pain scores or sample size over time, and very few studies used pregabalin as an active control arm, precluding assessment of the effect of this parameter on outcome.

A more recent meta-epidemiological study observed a decline effect in anti-depressants in 51 trials published between 1980 and 2011 [21]. Similar to our findings, there was a corresponding improvement in the placebo group. The authors proposed that it was due to high drop-out rates in the placebo groups of earlier trials, as disease severity scores at the time of dropout were carried forward and used in the analyses, an approach which may result in poorer outcome estimates in the placebo group, given the high rates of spontaneous clinical improvement. We found that attrition bias diminished over time, though we were unable to establish any statistical significance given the small sample size.

### Strengths & limitations

A major strength was the robustness of the study findings, as the decline in treatment effect was seen in all sensitivity and subgroup analyses (across dose and indications, where there were sufficient sample size). Further strengths include employing a rigorous definition of neuropathic pain, including RCTs only, including unpublished trials and including only indications for which pregabalin is recommended in the current NICE guidelines [2]. We used end-point pain scores rather than change scores as our outcome measure to reduce bias resulting from any baseline differences [50]. In fact, comparing end-point pain scores highlighted the small magnitude of difference between study groups. Our findings are broadly generalizable given that included trials were conducted in many countries, with participants aged between 41 and 72 years and evenly divided by sex.

This study has limitations. Strict inclusion criteria limited the sample size, therefore the study was underpowered to statistically assess risk of bias domains against publication year. We were therefore unable to determine whether improved pain reduction in placebo groups was due to methodological improvements in more recent trials. Second, few unpublished trials provided usable results for meta-analysis; however, most unpublished studies were conducted for regulatory purposes with similar results to published trials. Third, due to the scope of the study we did not assess other surrogate measures such as global impression of change, patient satisfaction, or adverse events. Lastly, the maximum trial duration was 17 weeks, thus the results of our study only apply to short-term use of pregabalin. The long-term effect of pregabalin treatment remains unstudied in clinical trials.

### Clinical relevance

Despite recent trials reporting low efficacy, pregabalin use is increasing. Any benefits of pregabalin should be interpreted in the context of MCID, which is accepted as a reduction of 1.7 points in pain severity on a 11-point scale [27–32]. The estimated effect of pregabalin treatment irrespective of dose, indication (except AHN) or time period ranged from 0.3 to 1.3 points, a level unlikely to provide clinically meaningful pain relief for patients.

## Conclusion

Our study provides evidence of a decline in the treatment effect of pregabalin relative to placebo over time. This finding was consistent across all doses and indications (except AHN), and it was not explained by changes in baseline differences or study characteristics in our study. The treatment effect of pregabalin is smaller than the accepted threshold of minimally clinically important difference. While causes of the decline are unclear, it likely reflects different populations and indications along with increasing use of concomitant treatments and improved study quality over time. This highlights the importance of applying caution to promising results in early studies of new interventions. In light of increasing prescription rates of pregabalin, clinicians should be wary of the impact of early results on clinical practice guidelines.

## Supporting information

S1 Table Search strategy. (PDF)

S2 Table Characteristics of included published studies. (PDF)

S3 Table Unpublished studies identifiers and access links. (PDF)

S4 Table Sensitivity analyses: Meta-regression results for treatment effect of pregabalin (points), per 5-year period (Sensitivity analyses 1 and 2). (PDF)

S5 Table Association between study characteristics and year. (PDF)

S6 Table Association between indication and year. (PDF)

S7 Table Cochrane risk of bias domains by 5-year period. (PDF)

S1 Fig Subgroup analysis 1: Forest plot of treatment effect of pregabalin by indication. (PDF)

S2 Fig Subgroup analysis 2: Forest plot of treatment effect of pregabalin by dose. (PDF)

S3 Fig Network meta-analysis of pregabalin doses. (PDF)

S4 Fig Sensitivity analysis 1: Forest plot of treatment effect of pregabalin by 5-year period, including unpublished* trials. (PDF)

S5 Fig Sensitivity analysis 1: Unadjusted meta-regression of treatment effect on year, including unpublished trials. (PDF)

S6 Fig Sensitivity analysis 2: Forest plot of treatment effect of pregabalin by 5-year period, including enriched enrolment studies. (PDF)

S7 Fig Sensitivity analysis 2: Unadjusted meta-regression of treatment effect on year, including enriched-enrolment trials. (PDF)

S8 Fig Sensitivity analysis 3: Forest plot of treatment effect of pregabalin by year (in quartiles). (PDF)

S9 Fig Cochrane risk of bias (low vs high/unclear risk) by domain and 5-year period. (PDF)

S10 Fig Subgroup analyses 3–7: Treatment effect of pregabalin by risk of bias (low risk vs high/unclear risk). (PDF)

S1 Dataset Pregabalin neuropathic pain dataset—Pain scores. (CSV)

S2 Dataset Pregabalin neuropathic pain dataset–Study characteristics. (CSV)

S3 Dataset Pregabalin neuropathic pain dataset—Risk of bias. (CSV)

S1 File Pregabalin neuropathic pain analysis code. (RMD)
