# Supplementary material for: A meta-epidemiological study on the reported treatment effect of pregabalin in neuropathic pain trials over time
Source: PLoS One. 2023 Jan 20;18(1):e0280593. doi: 10.1371/journal.pone.0280593 (PMC9858874; doi:10.1371/journal.pone.0280593)
Supplement: S1 Table — (PDF) [file pone.0280593.s001.pdf]

S1 Table. Search strategy

**Search terms**
1. PREGABALIN/<br>2. Pregabalin.tw<br>3. 1 or 2<br>4. Randomi.ab<br>5. Randomly.ab<br>6. Randomized controlled trial.pt<br>7. Controlled clinical trial.pt<br>8. Or/4-7<br>9. 3 and 8<br>10. Placebo.tw<br>11. 9 and 10
**Databases (published studies)**
MEDLINE (1946 – present)<br>EMBASE (1947 – present)<br>Cochrane Central Register of Controlled Trials
**Trial registers (unpublished studies)**
Australian New Zealand Clinical Trials Registry (*https://www.anzctr.org.au/*)<br><br>EU Clinical Trials Register (*https://www.clinicaltrialsregister.eu/*)<br><br>US National Library of Medicine ClinicalTrials.gov (https://clinicaltrials.gov/ct2/home)
