# Supplementary material for: A meta-epidemiological study on the reported treatment effect of pregabalin in neuropathic pain trials over time
Source: PLoS One. 2023 Jan 20;18(1):e0280593. doi: 10.1371/journal.pone.0280593 (PMC9858874; doi:10.1371/journal.pone.0280593)
Supplement: S2 Table — (PDF) [file pone.0280593.s002.pdf]

S2 Table. Characteristics of included published studies

Author	Year of publication	Country	N	Duration of treatment	Indication	Doses	Mean age (years)	Male %
**Published**								
Dworkin	2003	USA	172	8·0	PHN	600	71·5	46·8
Lesser	2004	USA	336	5·0	DPN	75, 300, 600	59·9	59·9
Rosenstock	2004	USA	144	8·0	DPN	300	59·7	56·2
Sabatowski	2004	Int'l	238	8·0	PHN	150, 300	72·1	45·0
Freynhagen	2005	Europe	338	12·0	mixed	600, flexi	62·2	54·1
Richter	2005	Int'l	243	6·0	DPN	150, 600	57·1	60·6
Siddall	2006	Australia	136	12·0	central NP	flexi	50·1	83·2
Van Seventer	2006	Int'l	366	13·0	PHN	150, 300, 600	70·7	45·7
Arezzo	2008	USA	167	13·0	DPN	600	58·3	61·7
Tolle	2008	Int'l	383	12·0	DPN	150, 300, 600	58·6	55·4
Vranken	2008	Netherlands	40	4·0	central NP	flexi	54·5	52·5
Moon	2010	Korea	238	8·0	mixed	flexi	60·2	46·3
Simpson	2010	USA and Puerto Rico	299	14·0	HIV	flexi	47·5	81·1
van Seventer	2010	Int'l	251	8·0	post-trauma	flexi	51·5	49·2
Ogawa	2010	Japan	369	13·0	PHN	150, 300, 600	70·1	53·4
Guan	2011	China	308	8·0	mixed	flexi	60·1	46·4

Author	Year of publication	Country	N	Duration of treatment	Indication	Doses	Mean age (years)	Male %
**Published (continued)**								
Kim	2011	Int'l	219	12·0	central NP	flexi	58·3	62·6
Satoh	2011	Japan	314	13·0	DPN	300, 600	61·4	75·5
Kanodia	2011	India	45	4.0	AHN	150	46.5	80.0
Jenkins	2012	Int'l	50	2·0	post-trauma	300	49·8	44·0
Mishra	2012	India	60	4·0	cancer	flexi	NA	NA
Cardenas	2013	Int'l	211	16·0	central NP	flexi	45·9	80·4
Rauck	2013	USA	186	13·0	DPN	300	59·2	57·5
Chappell	2014	Int'l	134	5·0	DPN	300	55·8	59·7
Smith	2014	Int'l	194	15·0	DPN	300	57·5	59·8
Vinik	2014	USA	158	5·0	DPN	300	60·0	52·4
Simpson	2014	Int'l	375	16·0	HIV	flexi	41·8	36·8
Holbech	2015	Denmark	138	5·0	mixed	300	59·3	59·4
Huffman	2015	Int'l	384	6·0	DPN	flexi	58·7	65·0
Malik	2015	USA	19	3·0	sciatica	300	43·1	47·4
Ziegler	2015	Int'l	122	6·0	DPN	300	59·3	54·5
Raskin	2016	Int'l	548	6·0	DPN	flexi	58·9	54·5
Liu	2017	China	220	8·0	PHN	300	64·9	54·0
Mathieson	2017	Australia	207	8·0	sciatica	flexi	53·8	44·4
Mu	2018	China	620	9·0	DPN	300	60·5	47·3
Markman	2018	Int'l	542	15·0	post-trauma	flexi	53·1	50·0
McDonnell	2018	USA	91	4·0	DPN	300	58·6	63·7
Baba	2020	Int'l	173	7·0	DPN	300	59·8	64·6

Author	Year of publication	Country	N	Duration of treatment	Indication	Doses	Mean age (years)	Male %
**Unpublished†**								
studyid=107	2014*	China	620	9·0	DPN	300	NA	47·3
studyid=109	2007*	Japan	369	13·0	PHN	150, 300, 600	70·1	53·4
studyid=118	2008*	Int'l	539	15·0	post-trauma	flexi	NA	50·9
studyid=110	2015*	Int'l	219	12·0	central NP	flexi	58·2	62·6
studyid=120	2007*	Korea	240	8·0	mixed	flexi	60·2	46·3
studyid=121	2014*	China	219	8·0	PHN	300	NA	54·1
studyid=123	2015*	USA	59	3·0	DPN	flexi	57·6	72·3
studyid=127	2008*	Australia	29	1·0	central NP	300	NA	86·7
**Enriched-enrolment**								
Baron	2010	Int'l	217	5·0	sciatica	flexi	52·5	47·9
Hewitt	2011	USA	103	2·7	mixed	flexi	59·8	56·7
Gilron	2011	Canada	157	5·0	mixed	flexi	59·5	46·5
Raskin	2014	Int'l	294	13·0	DPN	flexi	58·6	52·7
Gonzalez-Duarte	2016	Mexico	26	4·0	DPN	flexi	NA	NA
Huffman	2017	Int'l	413	13·0	PHN	flexi	62·0	38·0

Abbreviations: Int'l= international (multi-country); Pub year= year of publication; USA=United States

* For unpublished studies, the year of study end is used instead of year of publication.

† For trial identifiers and access links, see Supplement Table S7.
