# Supplementary material for: A meta-epidemiological study on the reported treatment effect of pregabalin in neuropathic pain trials over time
Source: PLoS One. 2023 Jan 20;18(1):e0280593. doi: 10.1371/journal.pone.0280593 (PMC9858874; doi:10.1371/journal.pone.0280593)
Supplement: S3 Table — (PDF) [file pone.0280593.s003.pdf]

S3 Table. Unpublished studies identifiers and access links.

Studyid	Identifier (ClinicalTrials.gov Identifier or EuDraCT Number)	Link
101	NCT00654940	https://ClinicalTrials.gov/show/NCT00654940
107	NCT01332149	https://ClinicalTrials.gov/show/NCT01332149
109	NCT00394901	https://ClinicalTrials.gov/show/NCT00394901
110	2012-003304-12	https://www.clinicaltrialsregister.eu/ctr-search/search?query=eudract_number:2012-003304-12
114	NCT01485094	https://ClinicalTrials.gov/show/NCT01485094
118	NCT00313820	https://ClinicalTrials.gov/show/NCT00313820
120	NCT00141219	https://ClinicalTrials.gov/show/NCT00141219
121	NCT01455428	https://ClinicalTrials.gov/show/NCT01455428
123	NCT01928381	https://ClinicalTrials.gov/show/NCT01928381
127	NCT00978341	https://ClinicalTrials.gov/show/NCT00978341
