# Supplementary material for: A meta-epidemiological study on the reported treatment effect of pregabalin in neuropathic pain trials over time
Source: PLoS One. 2023 Jan 20;18(1):e0280593. doi: 10.1371/journal.pone.0280593 (PMC9858874; doi:10.1371/journal.pone.0280593)
Supplement: S4 Table — (PDF) [file pone.0280593.s004.pdf]

S4 Table. Sensitivity analyses: Meta-regression results for treatment effect of pregabalin (points), per 5-year period (Sensitivity analyses 1 and 2)

Model	Pregabalin treatment effect size (points) per 5-year period (95% CI)
Sensitivity 1: Unpublished	
Unadjusted (effect of year alone)	-0·4 (-0·5 to -0·2)
Adjusted for indication, treatment duration (weeks) and study sample size	-0·4 (-0·5 to -0·2)
Sensitivity 2: Enriched enrolment studies	
Unadjusted (effect of year alone)	-0·4 (-0·5 to -0·2)
Adjusted for indication, treatment duration (weeks) and study sample size	-0·4 (-0·5 to -0·2)
