# Supplementary material for: A meta-epidemiological study on the reported treatment effect of pregabalin in neuropathic pain trials over time
Source: PLoS One. 2023 Jan 20;18(1):e0280593. doi: 10.1371/journal.pone.0280593 (PMC9858874; doi:10.1371/journal.pone.0280593)
Supplement: S5 Table — (PDF) [file pone.0280593.s005.pdf]

S5 Table. Association between study characteristics and year

Year	Correlation coefficient*	p-value
Sample size	-0·03	0·88
Treatment duration (weeks)	-0·1	0·42
Dose	-0·1	0·32

*Spearman's rank-order correlation
