# Supplementary material for: A meta-epidemiological study on the reported treatment effect of pregabalin in neuropathic pain trials over time
Source: PLoS One. 2023 Jan 20;18(1):e0280593. doi: 10.1371/journal.pone.0280593 (PMC9858874; doi:10.1371/journal.pone.0280593)
Supplement: S6 Table — (PDF) [file pone.0280593.s006.pdf]

S6 Table. Association between indication and year

	2001-2005	2006-2010	2011-2015	2016-2020	Total	p-value*
**Indication**						0·88
AHN	0 (0·0%)	0 (0·0%)	1 (6·2%)	0 (0·0%)	1 (2·6%)	
Cancer	0 (0·0%)	0 (0·0%)	1 (6·7%)	0 (0·0%)	1 (2·7%)	
Central NP	0 (0·0%)	2 (22·2%)	2 (13·3%)	0 (0·0%)	4 (10·8%)	
DPN	3 (50·0%)	2 (22·2%)	7 (46·7%)	4 (57·1%)	16 (43·2%)	
HIV	0 (0·0%)	1 (11·1%)	1 (6·7%)	0 (0·0%)	2 (5·4%)	
Mixed	1 (16·7%)	1 (11·1%)	2 (13·3%)	0 (0·0%)	4 (10·8%)	
PHN	2 (33·3%)	2 (22·2%)	0 (0·0%)	1 (14·3%)	5 (13·5%)	
Post-trauma	0 (0·0%)	1 (11·1%)	1 (6·7%)	1 (14·3%)	3 (8·1%)	
Sciatica	0 (0·0%)	0 (0·0%)	1 (6·7%)	1 (14·3%)	2 (5·4%)	

*Fisher's exact test
