# Supplementary material for: A meta-epidemiological study on the reported treatment effect of pregabalin in neuropathic pain trials over time
Source: PLoS One. 2023 Jan 20;18(1):e0280593. doi: 10.1371/journal.pone.0280593 (PMC9858874; doi:10.1371/journal.pone.0280593)
Supplement: S7 Table — (PDF) [file pone.0280593.s007.pdf]

S7 Table. Cochrane risk of bias domains by 5-year period

Risk of bias domain	Risk	2001-2005	2006-2010	2011-2015	2016-2020	Total	p-value[*]
Allocation concealment							0·72
	High or unclear	2 (33.3%)	2 (25.0%)	8 (50.0%)	3 (42.9%)	15 (40.5%)	
	Low	4 (66.7%)	6 (75.0%)	8 (50.0%)	4 (57.1%)	22 (59.5%)	
Blinding of outcome assessment							0·67
	High or unclear	3 (50.0%)	3 (37.5%)	9 (56.2%)	5 (71.4%)	20 (54.1%)	
	Low	3 (50.0%)	5 (62.5%)	7 (43.8%)	2 (28.6%)	17 (45.9%)	
Blinding of participants and personnel							0·13
	High or unclear	0 (0.0%)	2 (25.0%)	7 (43.8%)	4 (57.1%)	13 (35.1%)	
	Low	6 (100.0%)	6 (75.0%)	9 (56.2%)	3 (42.9%)	24 (64.9%)	
Funding source							0·91
	High or unclear	6 (100.0%)	7 (87.5%)	13 (81.2%)	6 (85.7%)	32 (86.5%)	
	Low	0 (0.0%)	1 (12.5%)	3 (18.8%)	1 (14.3%)	5 (13.5%)	
Incomplete outcome data							0·51
	High or unclear	3 (50.0%)	4 (50.0%)	6 (37.5%)	1 (14.3%)	14 (37.8%)	
	Low	3 (50.0%)	4 (50.0%)	10 (62.5%)	6 (85.7%)	23 (62.2%)	
Randomisation							0·81
	High or unclear	3 (50.0%)	2 (25.0%)	6 (37.5%)	2 (28.6%)	13 (35.1%)	
	Low	3 (50.0%)	6 (75.0%)	10 (62.5%)	5 (71.4%)	24 (64.9%)	
Selective reporting							-
	High or unclear	0 (0.0%)	0 (0.0%)	0 (0.0%)	0 (0.0%)	0 (0.0%)	
	Low	6 (100.0%)	8 (100.0%)	16 (100.0%)	7 (100.0%)	37 (100.0%)	

[*]Fisher's exact test
