# Supplementary figures and images for: A meta-epidemiological study on the reported treatment effect of pregabalin in neuropathic pain trials over time
Source: PLoS One. 2023 Jan 20;18(1):e0280593. doi: 10.1371/journal.pone.0280593 (PMC9858874; doi:10.1371/journal.pone.0280593)

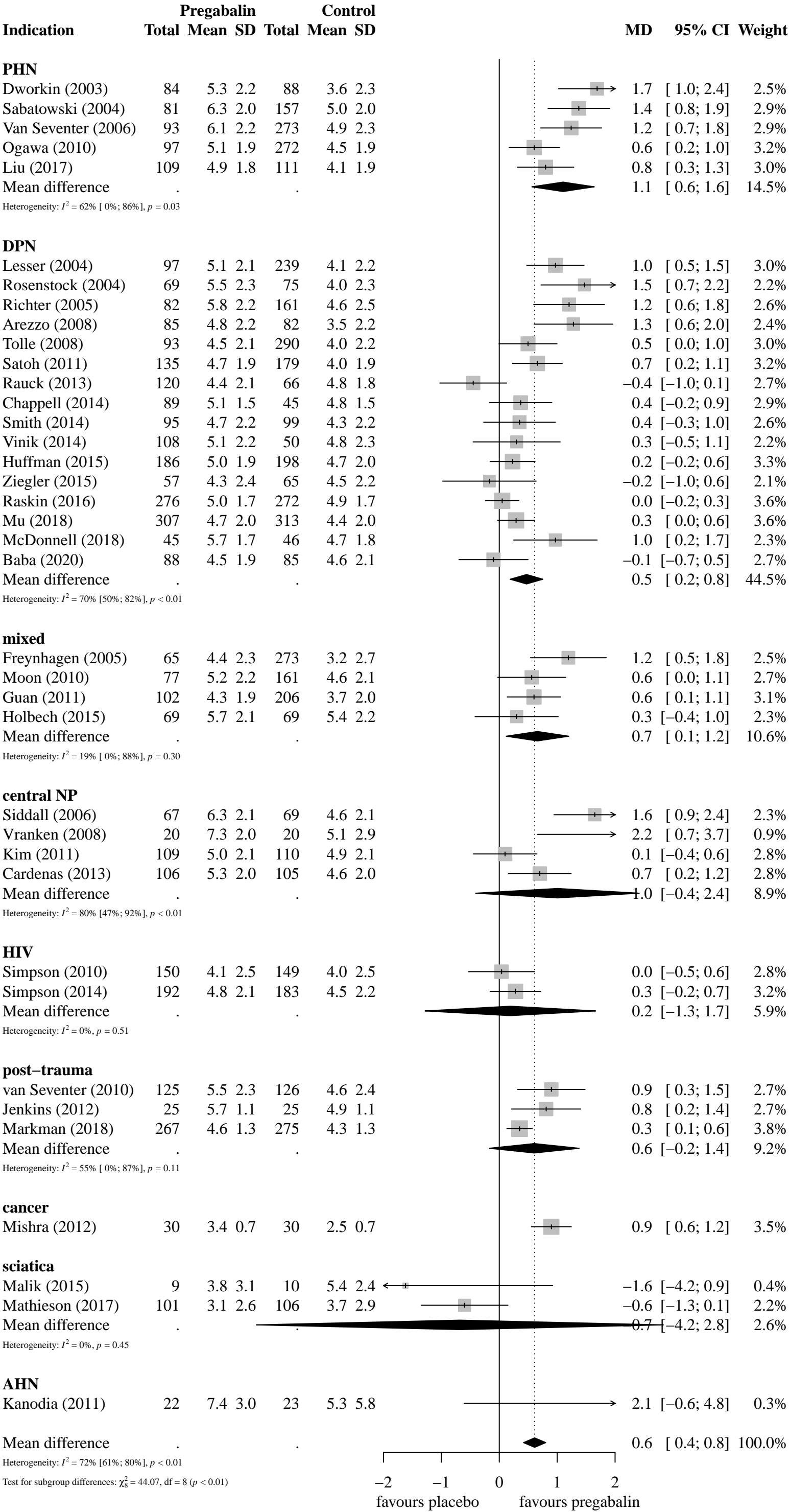

Supplement: S1 Fig — (PDF) [file pone.0280593.s008.pdf]

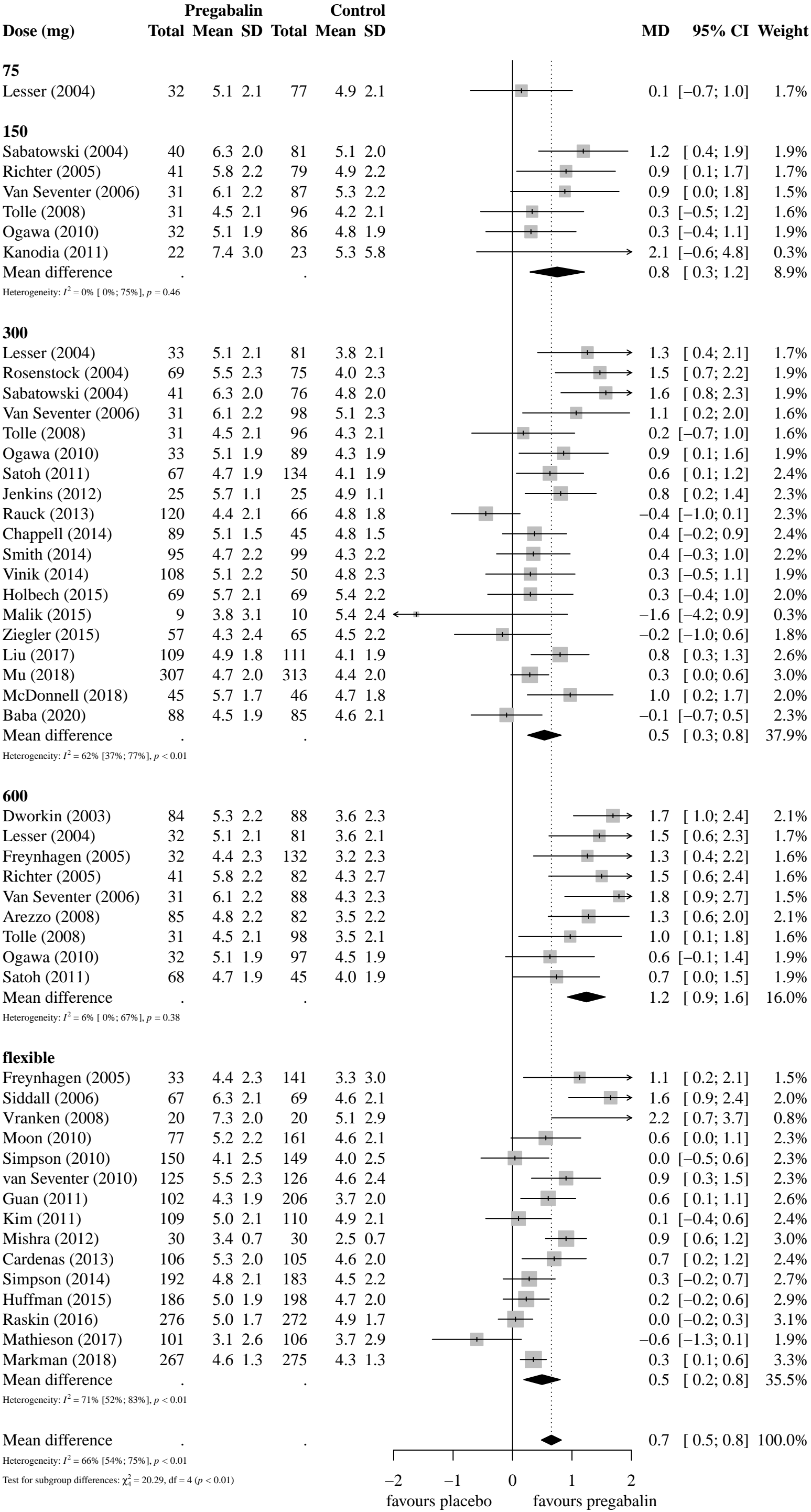

Supplement: S2 Fig — (PDF) [file pone.0280593.s009.pdf]

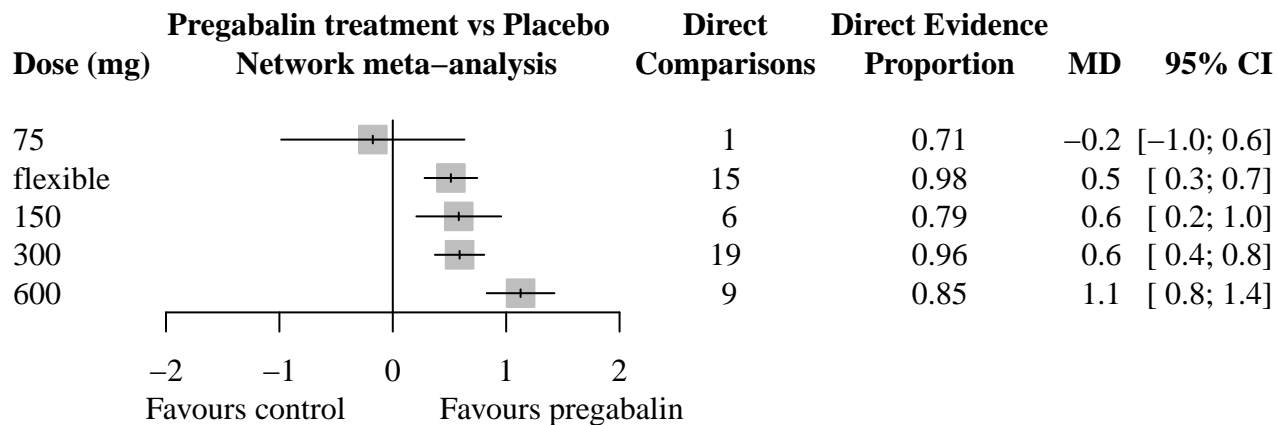

Supplement: S3 Fig — (PDF) [file pone.0280593.s010.pdf]

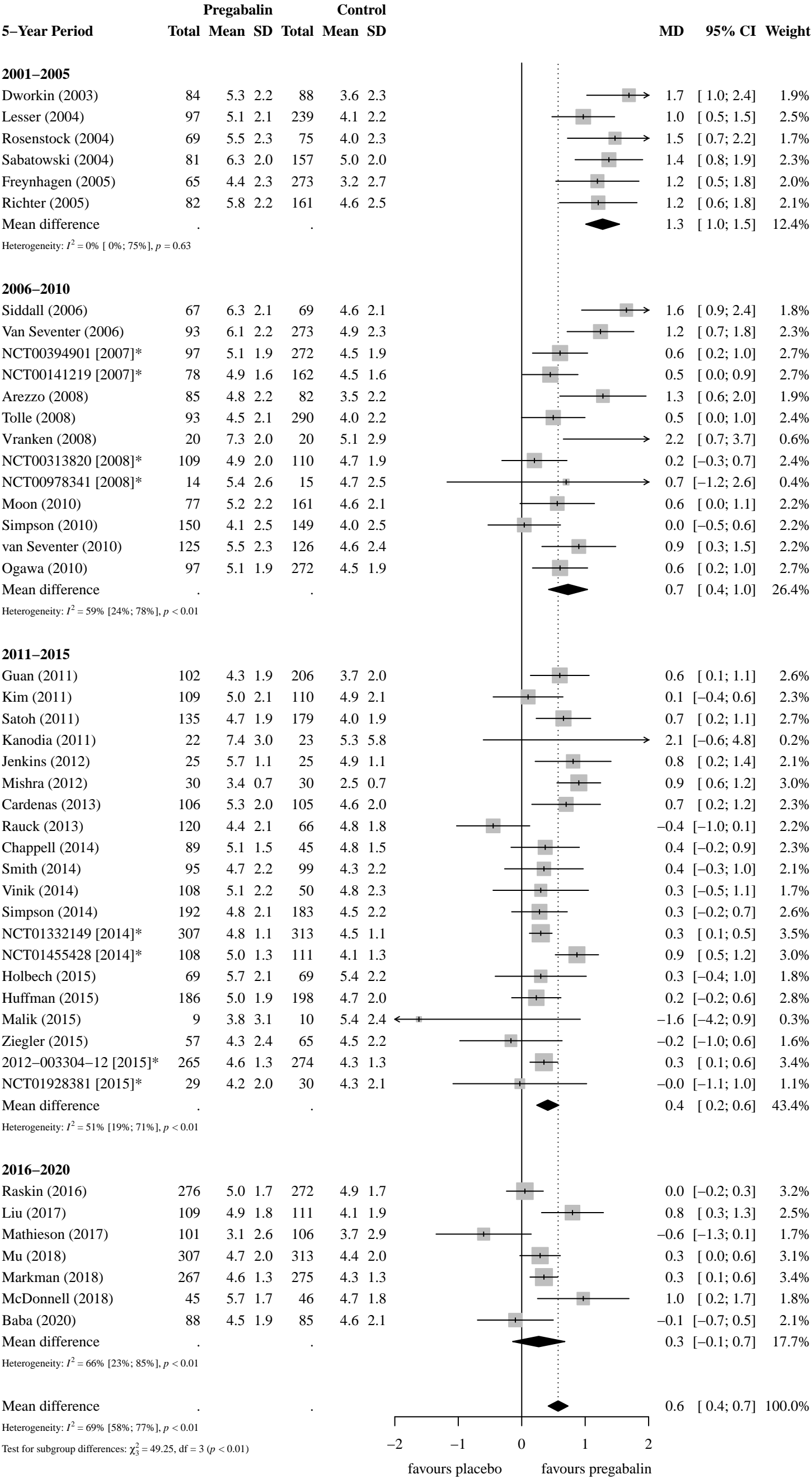

Supplement: S4 Fig — (PDF) [file pone.0280593.s011.pdf]

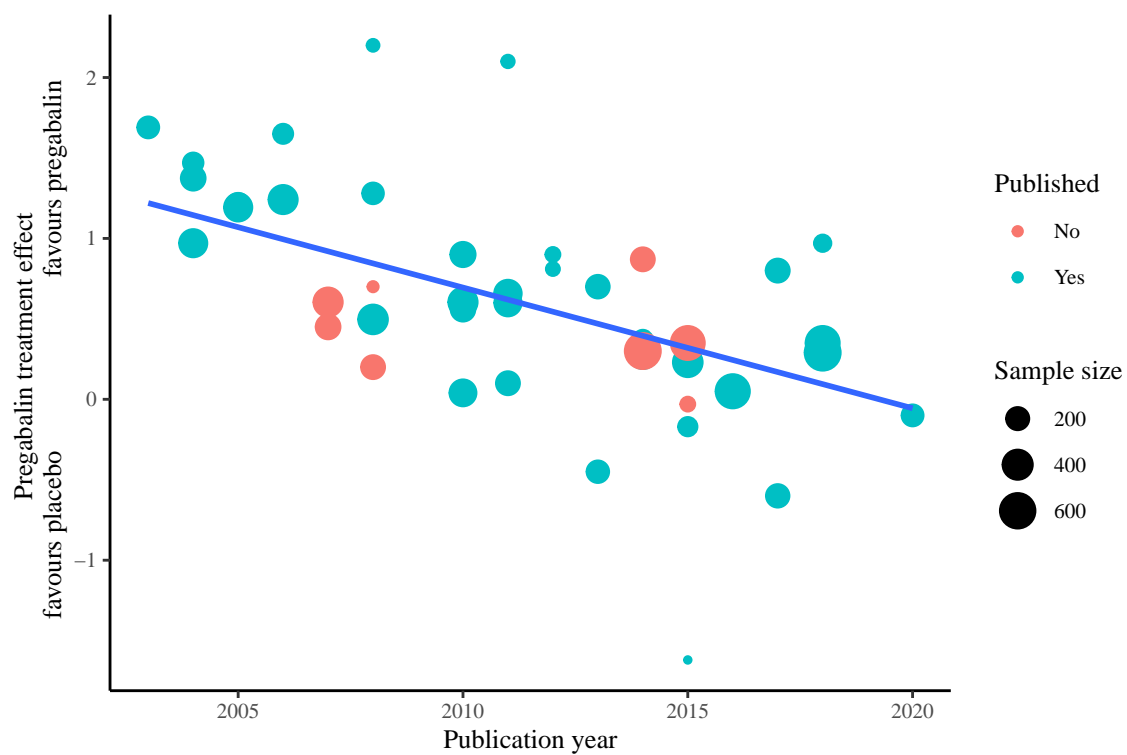

Supplement: S5 Fig — (PDF) [file pone.0280593.s012.pdf]

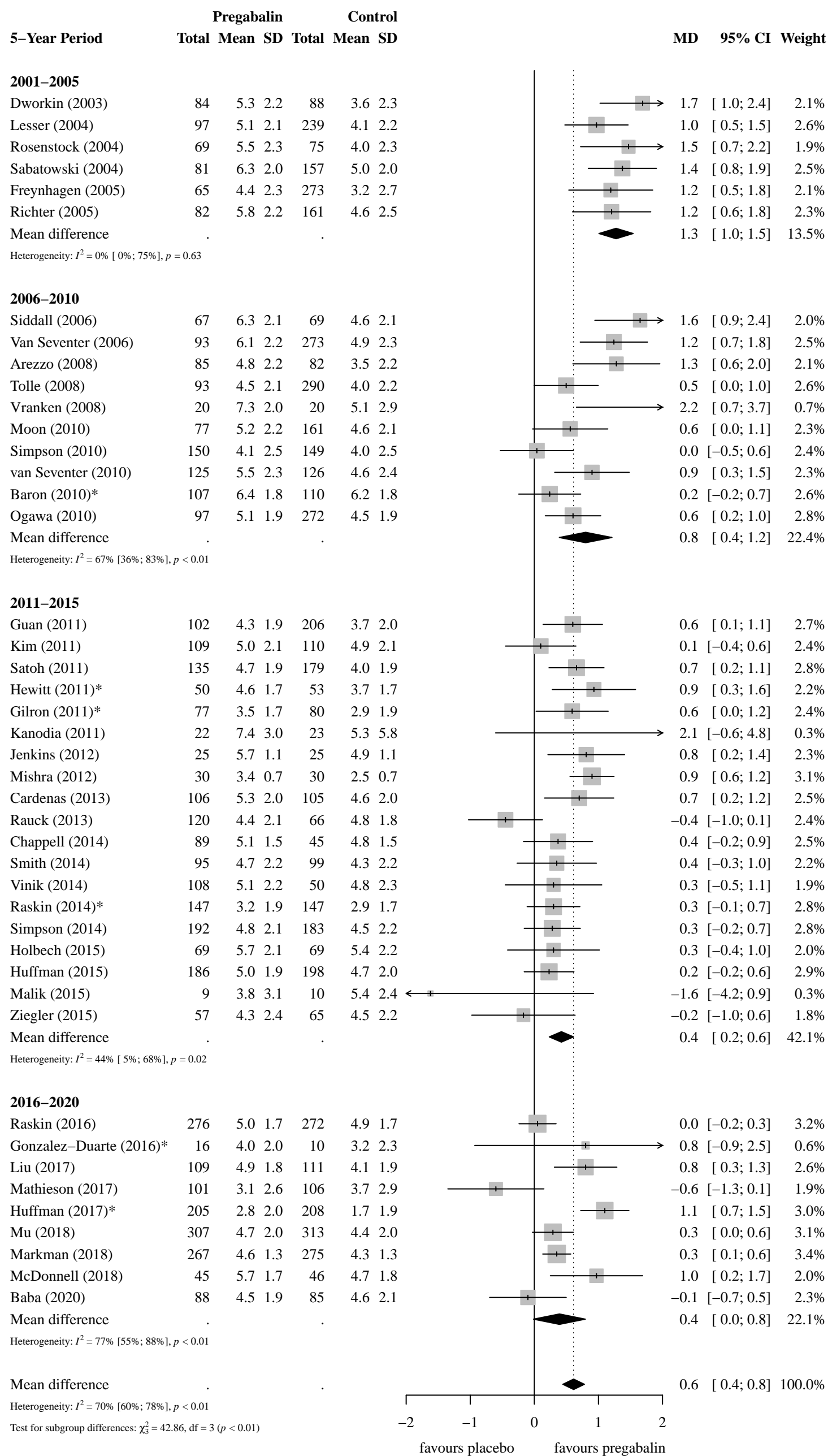

Supplement: S6 Fig — (PDF) [file pone.0280593.s013.pdf]

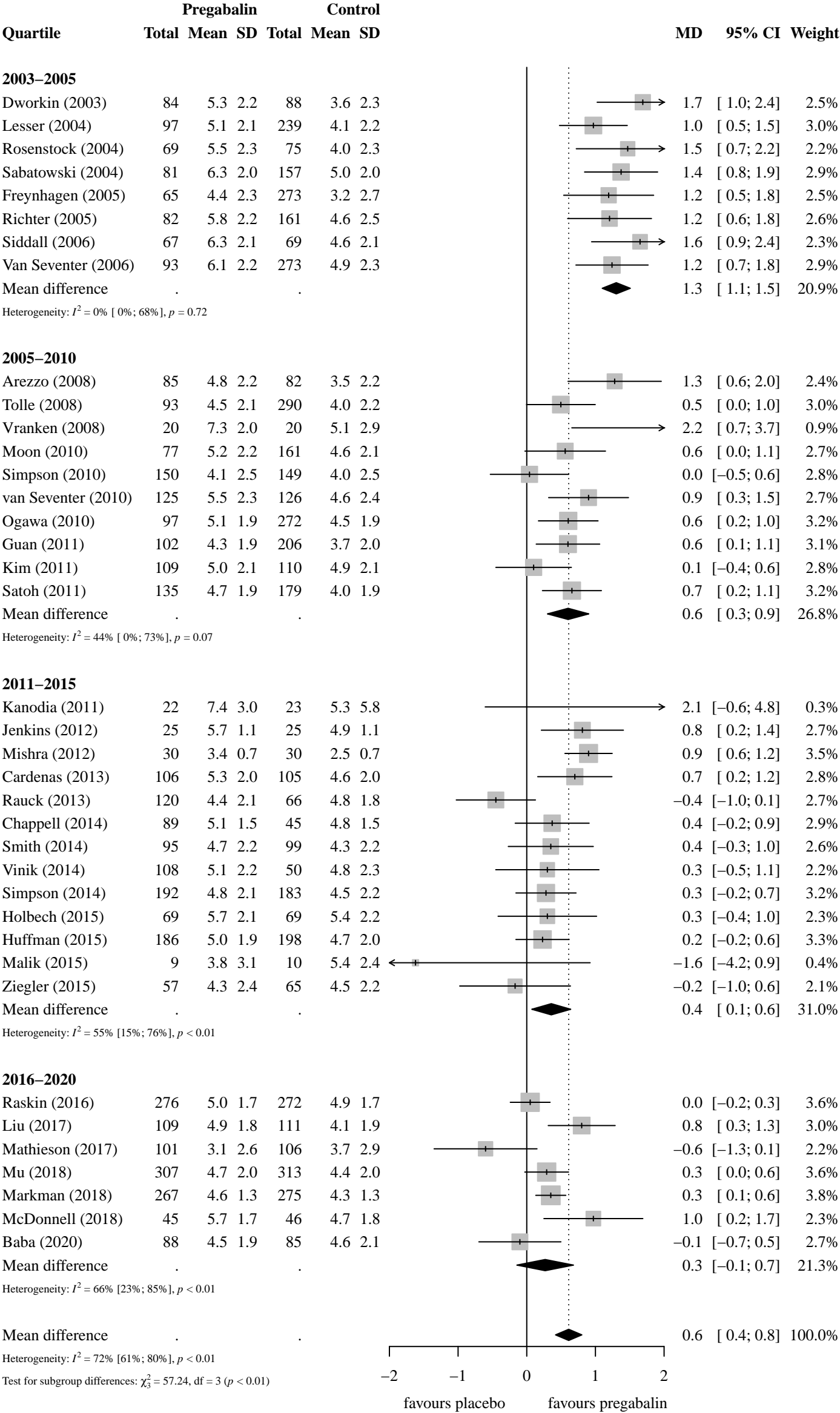

Supplement: S8 Fig — (PDF) [file pone.0280593.s015.pdf]

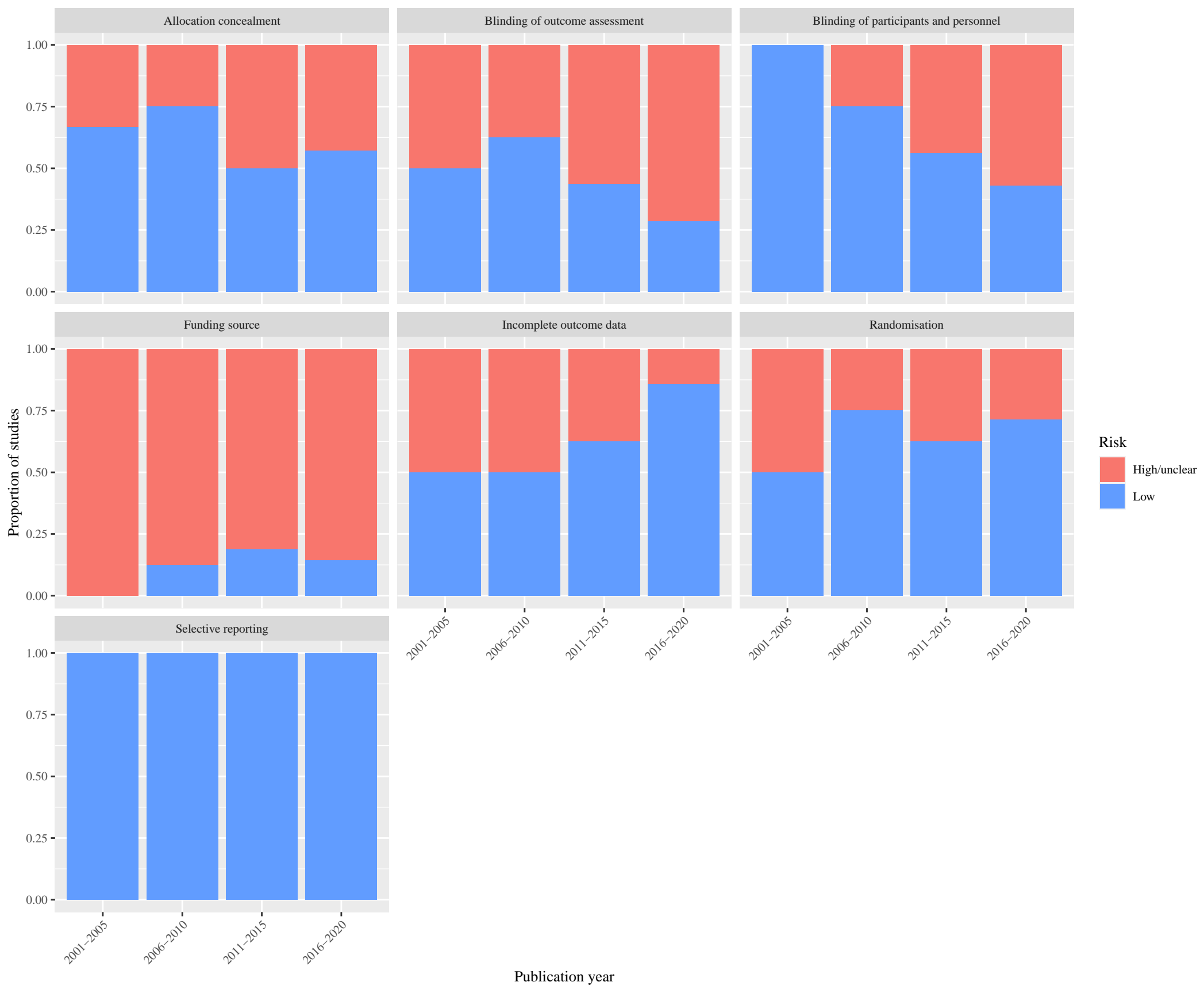

Supplement: S9 Fig — (PDF) [file pone.0280593.s016.pdf]
