# Supplementary material for: A meta-epidemiological study on the reported treatment effect of pregabalin in neuropathic pain trials over time
Source: PLoS One. 2023 Jan 20;18(1):e0280593. doi: 10.1371/journal.pone.0280593 (PMC9858874; doi:10.1371/journal.pone.0280593)
Supplement: S7 Fig — (PDF) [file pone.0280593.s014.pdf]

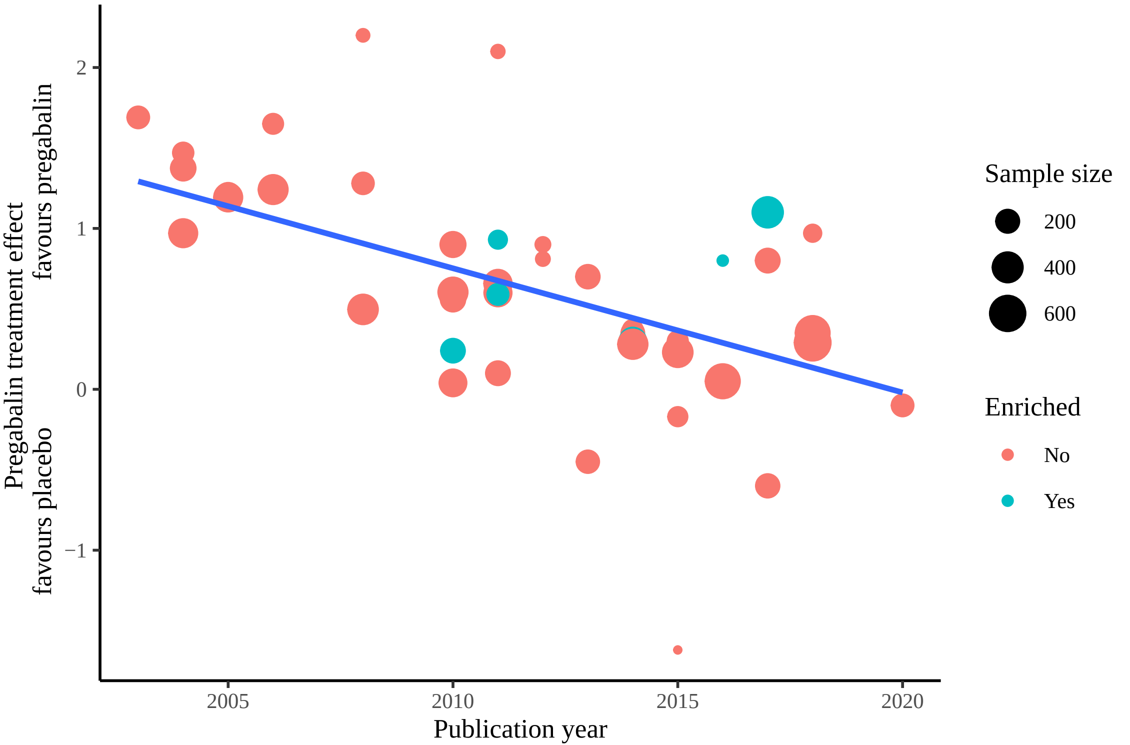
Pregabalin treatment effect
favours placebo
favours pregabalin
2
1
0
−1
2005
2010
2015
2020
Publication year
Sample size
200
400
600
Enriched
No
Yes
