# Supplementary material for: A meta-epidemiological study on the reported treatment effect of pregabalin in neuropathic pain trials over time
Source: PLoS One. 2023 Jan 20;18(1):e0280593. doi: 10.1371/journal.pone.0280593 (PMC9858874; doi:10.1371/journal.pone.0280593)
Supplement: S10 Fig — (PDF) [file pone.0280593.s017.pdf]

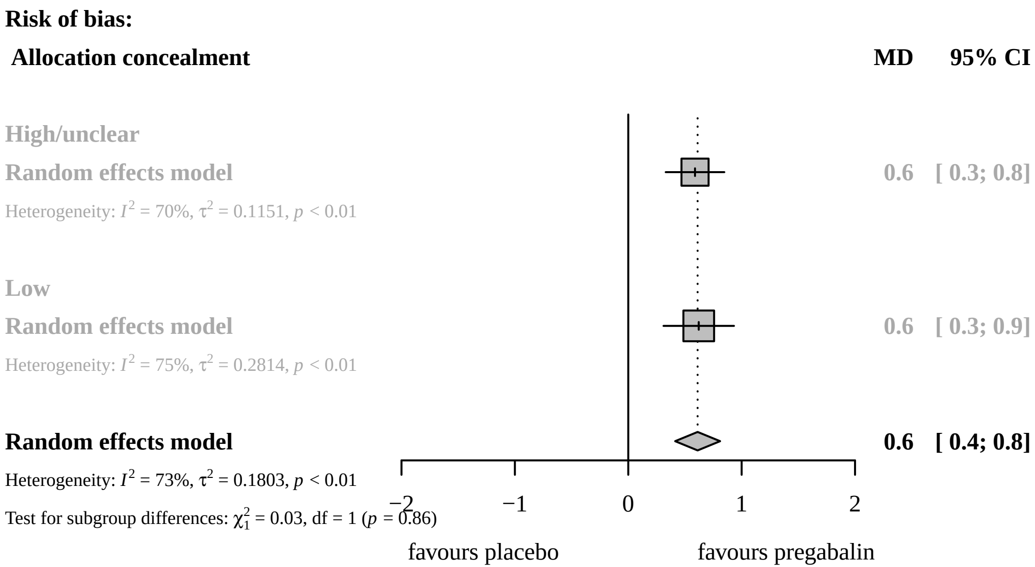

Risk of bias:
Allocation concealment
MD
95% CI
High/unclear
Random effects model
0.6
[0.3; 0.8]
Heterogeneity: I^2 = 70%, τ^2 = 0.1151, p < 0.01
Low
Random effects model
0.6
[0.3; 0.9]
Heterogeneity: I^2 = 75%, τ^2 = 0.2814, p < 0.01
Random effects model
0.6
[0.4; 0.8]
Heterogeneity: I^2 = 73%, τ^2 = 0.1803, p < 0.01
Test for subgroup differences: χ^2_1 = 0.03, df = 1 (p = 0.86)
−2
−1
0
1
2
favours placebo
favours pregabalin

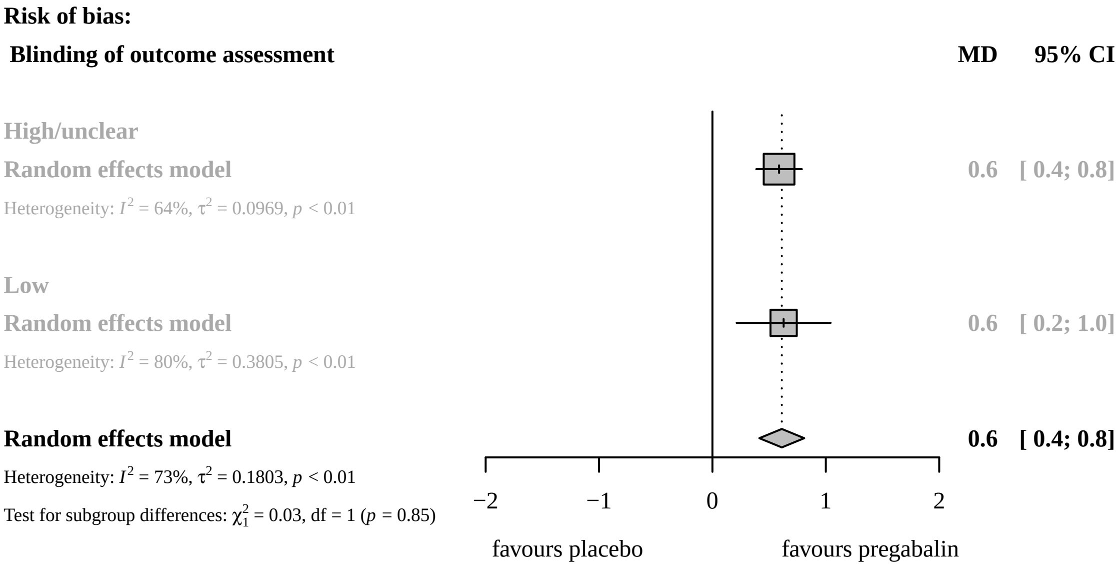
Risk of bias:
Blinding of outcome assessment
MD
95% CI
High/unclear
Random effects model
0.6
[0.4; 0.8]
Heterogeneity: I^2 = 64%, τ^2 = 0.0969, p < 0.01
Low
Random effects model
0.6
[0.2; 1.0]
Heterogeneity: I^2 = 80%, τ^2 = 0.3805, p < 0.01
Random effects model
0.6
[0.4; 0.8]
Heterogeneity: I^2 = 73%, τ^2 = 0.1803, p < 0.01
Test for subgroup differences: χ^2_1 = 0.03, df = 1 (p = 0.85)
−2
−1
0
1
2
favours placebo
favours pregabalin

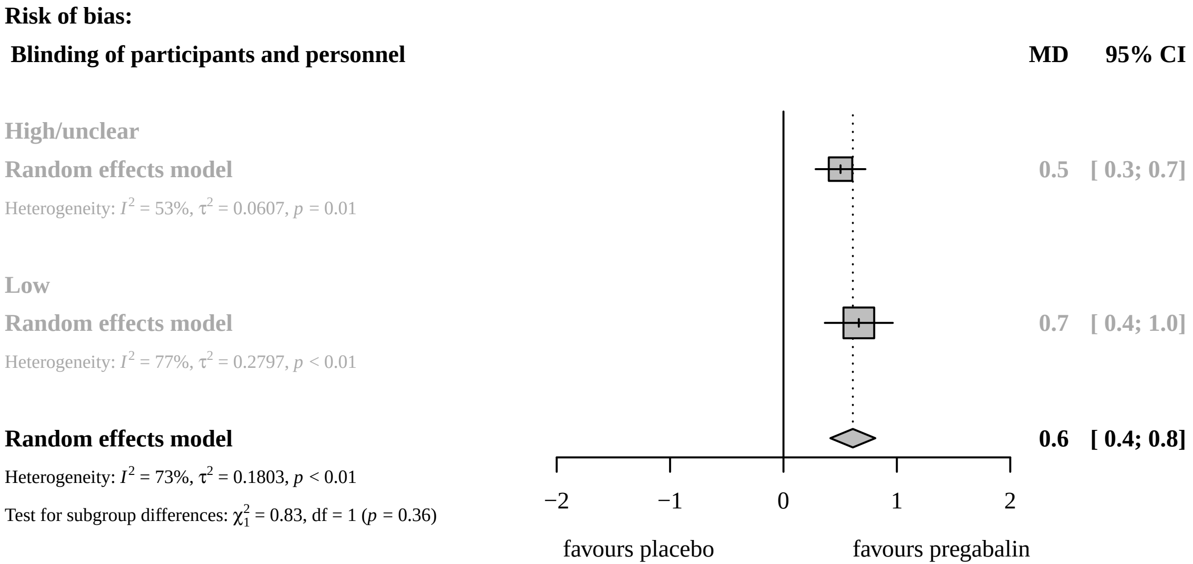
Risk of bias:
Blinding of participants and personnel
MD
95% CI
High/unclear
Random effects model
0.5
[0.3; 0.7]
Heterogeneity: I^2 = 53%, τ^2 = 0.0607, p = 0.01
Low
Random effects model
0.7
[0.4; 1.0]
Heterogeneity: I^2 = 77%, τ^2 = 0.2797, p < 0.01
Random effects model
0.6
[0.4; 0.8]
Heterogeneity: I^2 = 73%, τ^2 = 0.1803, p < 0.01
Test for subgroup differences: χ^2_1 = 0.83, df = 1 (p = 0.36)
−2
−1
0
1
2
favours placebo
favours pregabalin

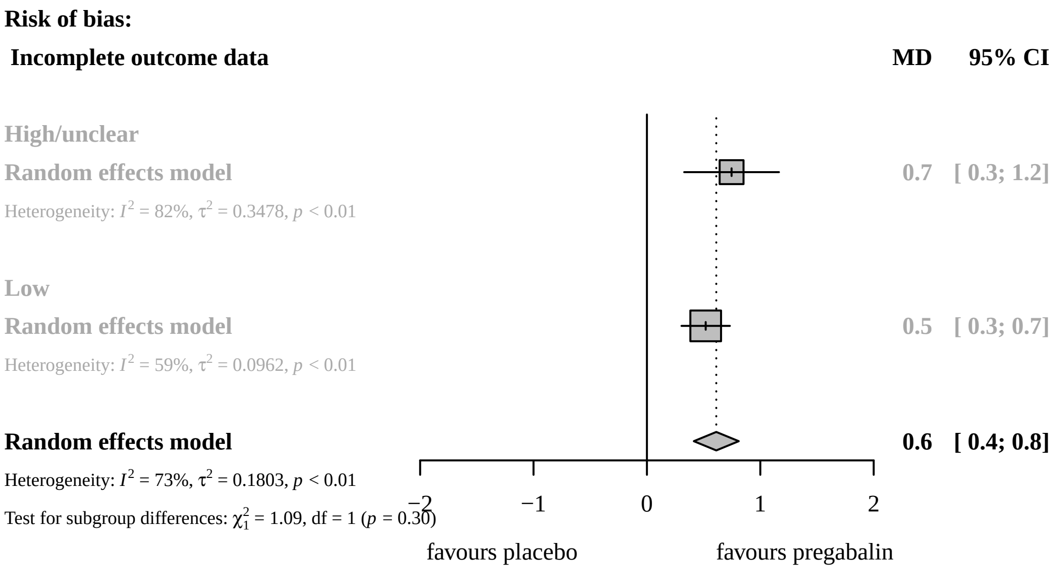
Risk of bias:
Incomplete outcome data
MD
95% CI
High/unclear
Random effects model
0.7
[0.3; 1.2]
Heterogeneity: I^2 = 82%, τ^2 = 0.3478, p < 0.01
Low
Random effects model
0.5
[0.3; 0.7]
Heterogeneity: I^2 = 59%, τ^2 = 0.0962, p < 0.01
Random effects model
0.6
[0.4; 0.8]
Heterogeneity: I^2 = 73%, τ^2 = 0.1803, p < 0.01
Test for subgroup differences: χ^2_1 = 1.09, df = 1 (p = 0.30)
−2
−1
0
1
2
favours placebo
favours pregabalin

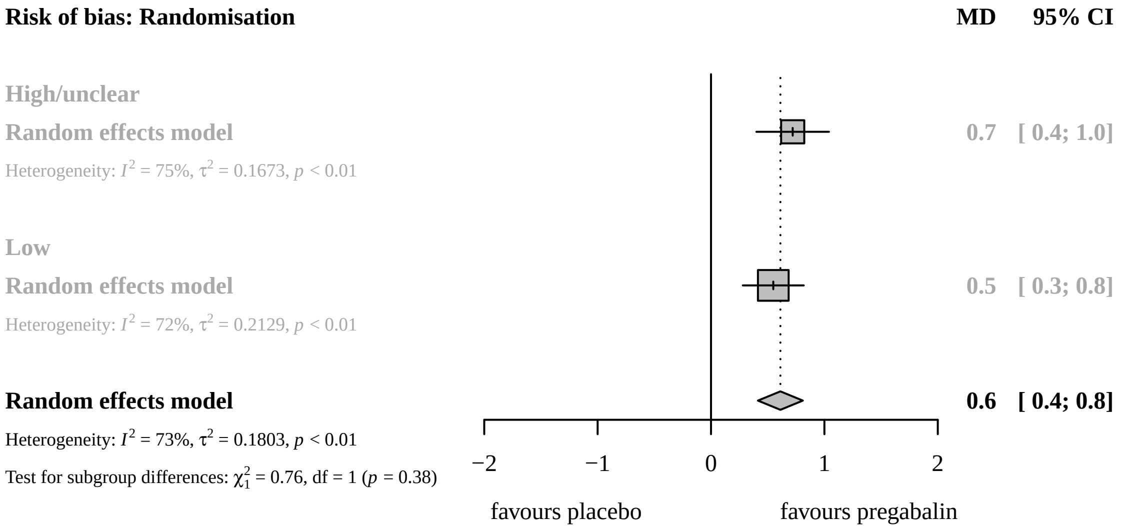
Risk of bias: Randomisation
MD
95% CI
High/unclear
Random effects model
0.7
[0.4; 1.0]
Heterogeneity: $I^2 = 75\%$, $\tau^2 = 0.1673$, $p < 0.01$
Low
Random effects model
0.5
[0.3; 0.8]
Heterogeneity: $I^2 = 72\%$, $\tau^2 = 0.2129$, $p < 0.01$
Random effects model
0.6
[0.4; 0.8]
Heterogeneity: $I^2 = 73\%$, $\tau^2 = 0.1803$, $p < 0.01$
Test for subgroup differences: $\chi^2_1 = 0.76$, df = 1 ($p = 0.38$)
−2
−1
0
1
2
favours placebo
favours pregabalin
